# Comparison of miRNA Expression Profiles between HIV-1 and HIV-2 Infected Monocyte-Derived Macrophages (MDMs) and Peripheral Blood Mononuclear Cells (PBMCs)

**DOI:** 10.3390/ijms21186970

**Published:** 2020-09-22

**Authors:** Santanu Biswas, Emily Chen, Mohan Haleyurgirisetty, Sherwin Lee, Indira Hewlett, Krishnakumar Devadas

**Affiliations:** Laboratory of Molecular Virology, Division of Emerging and Transfusion Transmitted Diseases, Center for Biologics Evaluation and Research, Food and Drug Administration, 10903 New Hampshire Avenue, Silver Spring, MD 20993-0002, USA; Santanu.Biswas@fda.hhs.gov (S.B.); emilyy314@gmail.com (E.C.); Mohan.Haleyurgirisetty@fda.hhs.gov (M.H.); Sherwin.Lee@fda.hhs.gov (S.L.)

**Keywords:** monocyte-derived macrophages (MDMs), peripheral blood mononuclear cells (PBMCs), HIV-1, HIV-2, microRNA

## Abstract

During the progression of HIV-1 infection, macrophage tropic HIV-1 that use the CCR5 co-receptor undergoes a change in co-receptor use to CXCR4 that is predominately T cell tropic. This change in co-receptor preference makes the virus able to infect T cells. HIV-2 is known to infect MDMs and T cells and is dual tropic. The aim of this study was to elucidate the differential expression profiles of host miRNAs and their role in cells infected with HIV-1/HIV-2. To achieve this goal, a comparative global miRNA expression profile was determined in human PBMCs and MDMs infected with HIV-1/HIV-2. Differentially expressed miRNAs were identified in HIV-1/HIV-2 infected PBMCs and MDMs using the next-generation sequencing (NGS) technique. A comparative global miRNA expression profile in infected MDMs and PBMCs with HIV-1 and HIV-2 identified differential expression of several host miRNAs. These differentially expressed miRNAs are likely to be involved in many signaling pathways, like the p53 signaling pathway, PI3K-Akt signaling pathways, MAPK signaling pathways, FoxO signaling pathway, and viral carcinogenesis. Thus, a comparative study of the differential expression of host miRNAs in MDMs and T cell in response to HIV-1 and HIV-2 infection will help us to identify unique biomarkers that can differentiate HIV-1 and HIV-2 infection.

## 1. Introduction

The Human Immunodeficiency Virus (HIV) is an RNA virus belonging to the genera of lentiviruses, a family of retrovirus that is characterized by its chronic and persistent infection. HIV can be further classified into two major types, HIV-1 and HIV-2. HIV-1 and HIV-2 are characterized by a high degree of genetic variation. These viruses closely share common pathways involved in viral transmission, replication, and pathogenesis. Although these two viruses share many similar characteristics, major differences exist between them. HIV-1 is more virulent, with higher levels of circulating virus than HIV-2. Reports have indicated that HIV-2 infected patients who progress to AIDS live for a longer time and have relatively higher CD4 counts compared to HIV-1 infected patients who progress to AIDS. Furthermore, progression to immunodeficiency is less frequent with HIV-2 infection than with HIV-1 infection, even though the minority who do progress cannot be distinguished clinically from HIV-1 infected patients. HIV-1 is more pathogenic than HIV-2, with higher measurable levels of plasma viremia. However, the exact mechanisms contributing to these differences is not completely understood.

The biology and pathogenesis of HIV infection has been largely studied since the discovery of the virus in 1983. During unprotected sexual intercourse, HIV-1 is mainly transmitted through genital and/or rectal mucosal sites, resulting in the rapid infection of target cells and is widely disseminated through the bloodstream to lymph nodes and other organs including the gut, spleen, lungs, and the brain [1]. The detection of CCR5-utilizing “macrophage-tropic” forms of HIV, essentially in the plasma of all newly infected individuals, is consistent with the notion that macrophages are among the cells that are initially infected upon exposure to HIV [2] and represent an early target for HIV infection [3,4,5,6]. Furthermore, macrophages are capable of transmitting HIV to other macrophages or T cells via cell–cell contact [7]. The HIV-infected T cells are able to migrate within lymph nodes, further disseminating HIV by a cell-to-cell transfer mechanism [8]. For instance, T cell-to-T cell and macrophage-to-T cell transfer of the virus is an efficient way for the virus to spread locally [9,10,11,12,13]. While T cells are known to be rapidly depleted after infection in patients, macrophages in contrast are able to survive infection and are able to migrate into all body tissues, further spreading the virus. HIV-1-infected macrophages are characteristically resistant to virus-mediated killing, functioning as an HIV-1 reservoir [14]. CD8+ cytotoxic T lymphocytes (CTLs) control viral levels during acute and chronic stages of HIV infection and reduce the progression of disease caused by HIV [15,16]. Most studies have focused on the control of infected CD4+ T cells by CTLs, with less focus on infected macrophages. A few in vitro studies have shown that HIV-specific CTLs can eliminate HIV-infected macrophages [17,18,19,20]. The majority (up to 90%) of newly transmitted HIV-1 uses the CCR5 coreceptor. R5 virus, also known as macrophage (M)-tropic virus due to its ability to infect macrophages, and X4-tropic virus, also known as T-tropic virus, emerge in about 50–60% of infected individuals with an average time to emergence of 5 years. X4-tropic virus is associated with more pronounced depletion of CD4 T cells than R5 virus [21,22].

During the progression of HIV-1 infection, M-tropic HIV-1 that uses the CCR5 co-receptor undergoes a change in co-receptor use to CXCR4 that is predominately T-cell-tropic. Thus, during progression of HIV-1 infection, there is a change in tropism or a class switch in co-receptor usage. This change in co-receptor preference makes the virus able to infect T cells. Therefore, it is important to study the two cellular compartments MDMs and T cells infected by HIV-1 virus and identify differential expression of host factors in MDMs and T cells in response to HIV-1 infection. HIV-2 is known to infect MDMs and T cells and is dual tropic. Not much is known about co-receptor usage during HIV-2 infection. Thus, a comparative study of the differential expression of host miRNAs in MDMs and T cell in response to HIV-1 and HIV-2 infection will help us to identify unique biomarkers that can differentiate HIV-1 and HIV-2 infection. Evaluating the differential expression of host miRNAs in macrophages as well as T cells will help us to understand co-receptor use and cell tropism going from CCR5 (macrophage tropism) to CXCR4 (T cell line tropism). Identifying differentially expressed miRNAs between these two cell types could lead us to discover biomarkers that can detect individuals in the early stage of HIV infection.

Revealing the mechanisms of host responses to infection is particularly important for the prevention and treatment of HIV. However, important insights have been achieved within the last decade, notably by extensive functional genomic studies [23,24,25,26,27]. Previous studies have reported that gene expression profiling analysis in HIV-1 and HIV-2 infected PBMCs identified many differentially expressed genes involved in cellular metabolism, apoptosis, B and T cell activation and proliferation, cellular receptors, and transcription factors in HIV-1 infected PBMCs; genes for proteins like heat shock protein (HSPA6), splicing factor, arginine/serine-rich 9 (SFRS9), and keratin (KRT1) were differentially expressed in HIV-2 infected PBMCs [28]. Furthermore, genes involved in glutathione metabolism and lysine degradation were differentially regulated only in HIV-1 infected MDMs. In HIV-2 infected MDMs, cullin-2 (CUL 2), splicing factor, arginine/serine-rich 9 (SFRS9), and Retinoblastoma-Binding Protein 4 (RBBP4) genes were differentially expressed [29]. However, the molecular mechanisms explaining such differences in cellular gene expression upon HIV infection remain unknown. Gene expression can be controlled by different key processes, including post-transcriptional regulation by microRNAs (miRNAs), which are small RNA molecules from 21 to 23 nt. These non-coding small RNAs specifically bind to functional elements located in the 3′ UTR of mRNA targets and repress gene expression, either by inhibiting translation or promoting mRNA degradation through the Dicer pathway [30]. Numerous reports have shown that miRNAs also play an important role in virus–host interactions, particularly in the immune response to infections [31]. Recent reports on different viruses like prototype foamy virus (PFV)-1, vaccinia virus (VV), human immunodeficiency virus (HIV)-1, and hepatitis C virus (HCV), suggest that cellular miRNAs can modulate infection by directly targeting viral genes [32,33,34,35]. Thus, variation in miRNA expression upon viral infection can contribute to the cellular response by targeting both cellular and viral genes. Similarly, it was found that the expression pattern of miRNAs in PBMCs, CD8+ T cells, monocytes, and CD4+ T cells from HIV-1-infected subjects were not the same [32,36,37,38,39]. Wang et al. reported that miR-223 and miR-28 showed different expression levels between monocytes and macrophages in HIV infection [37]. Ahluwalia et al. showed that miR-29a in the PBMC and CD4+ T cells can reduce viral replication [40]. Other studies have shown that five miRNAs, including miR-28 and miR-223, can directly target to the 3′ end of HIV mRNAs in order to inhibit viral mRNA expression in CD4+ T cells [32]. 

The existing antiretroviral drugs used in highly active antiretroviral therapies (HAART) were identified and characterized in T cell cultures. In a recent study, various clinically relevant HIV drugs were found to display significantly lower intracellular concentrations in macrophages versus lymphocytes, leading to a 5 to 200 times difference in antiviral potency between the two cell types [41]. HAART has dramatically improved the survival of HIV-positive patients, generally by reversing complications from T cell loss. However, several major diseases such as atherosclerosis [42], HIV-associated neurocognitive disorder [43], metabolic diseases, and cancer [44] are becoming increasingly prevalent in HAART-treated patients [45]. These diseases result from persistent macrophage activation and currently affect more than half of HIV-infected individuals [46], suggesting a failure in the primarily T cell-targeted approach to treat HIV-associated diseases. In addition, in the macrophage compartment, the integrated viral genome, upon reactivation, may serve as a relatively drug resistant, long-term source of HIV. Furthermore, with the emergence of cure-related treatments, there is an urgent need to identify host-based targets that are cell type (macrophages or T cells)-specific.

MiRNAs have been shown to be involved in HIV replication, but only a few studies have further investigated this aspect in a human cellular model infected with human immunodeficiency viruses. Here, we performed global miRNA profiling in MDMs and PBMCs infected by the two different types of human immunodeficiency virus (HIV-1 and HIV-2) to elucidate related pathways or candidates that may serve as therapeutic targets for HIV-1 and HIV-2. In this study, host miRNA expression profiles were analyzed using the next-generation sequencing (NGS) technique. Differentially expressed miRNAs were identified in HIV-1/HIV-2-infected MDMs and PBMCs. Analysis of the comparative global miRNA expression profile in MDMs infected with HIV-1 and HIV-2 identified differential expression of several host miRNAs (hsa-let-7g-3p, hsa-let-7i-3p, hsa-miR-7-1-3p, hsa-miR-331-3p, hsa-miR-375, hsa-miR-26a-2-3p, and has-miR-4286). In HIV-1-infected PBMCs, 41 miRNAs were upregulated and 97 miRNAs were downregulated compared to uninfected control cells. Only four miRNAs were upregulated in the HIV-2-infected PBMCs compared to uninfected control cells. Putative functional targets of these differentially expressed miRNAs include several genes involved in the p53 signaling pathway, PI3K-Akt signaling pathways, MAPK signaling pathways, the FoxO signaling pathway, and viral carcinogenesis.

## 2. Results

### 2.1. Length Distribution and Annotation of Small RNAs

RNA samples from PBMCs isolated from three independent donors productively infected with HIV-1 MN or HIV-2 ROD (Table 1) were used to obtain miRNA expression data from NGS. Similarly, MDMs were isolated from three independent donors and infected with HIV-1 BaL or HIV-2 ROD (Table 1). We have also checked the cytopathic effects of HIV-1 BaL or HIV-1 MN after 7 days post infection. Eighteen small RNA libraries were made using RNA isolated from MDMs and PBMCs infected with HIV-1 or HIV-2 and uninfected controls isolated from three independent donors. The libraries were sequenced simultaneously using Illumina HiSeq 2000. High quality sequence reads were acquired from the total RNAs isolated from HIV-1- and HIV-2-infected MDMs and PBMCs. For HIV-1-infected MDMs, more than 1.2 million clean reads and for HIV-1-infected PBMCs, more that 10 million clean reads were acquired. Similarly, greater than 1.2 million and 14 million clean reads were acquired from HIV-2-infected MDMs and PBMCs, respectively. More than 1.25 million and 10 million high-quality sequence reads were obtained in all MDM and PBMC controls. After removing the adaptor sequences, low-quality reads and contaminated reads in each of the small RNA libraries, a total of >1 million read and >10 million small RNA trimmed reads were collected from all MDM and PBMC samples, respectively, with the exception of one uninfected control sample (Table 2). In all of the libraries, except for one control MDM, the proportion of trimmed reads was greater than 75%. The length distribution of small RNAs in all sixteen libraries that were used for sequencing was determined to be 16–30 nt long. In fifteen libraries, the length distribution of clean sequences was determined to be >15 nt, and out of these, the 22 nt class of small RNAs were the most abundant—22.34% in HIV-1-infected MDMs and 32.60% in the HIV-2-infected PBMCs (Figure S1). In the one remaining library from MDM donor# 3, the most abundant class of small RNAs was determined to be 23 nt long. 

Only the high-quality clean reads (length > 15 nt) were included for further analysis. The unique reads were mapped to the human genome using the Rfam database. The genome-matched reads of each of the libraries were annotated and classified into different categories of RNAs (Figure S2), including miRNA, snRNAs, tRNAs, rRNAs, and other noncoding RNAs (sed_pseudogene, and repeat-associated small RNAs). As shown in Figure S2A, the proportion of miRNA sequences was 48–55% in control (three libraries), 62–73% in HIV-1 (three libraries), and 64–74% in HIV-2 (three libraries)-infected MDMs libraries. The miRNAs sequencing reads seem to be low in three control libraries in MDMs. In Figure S2B, the proportion of miRNA sequences were 61–71% in control (three libraries), 46–58% in HIV-1 (three libraries), and 74–75% in HIV-2 (three libraries)-infected PBMCs. As shown in Figure S2, the proportion of rRNA sequences (<15%) was low and no mRNA degradation fragments were found in all of the eighteen libraries. This indicates the high quality and high credibility of the sequencing samples.

### 2.2. Identification of miRNAs

**Figure 1:**
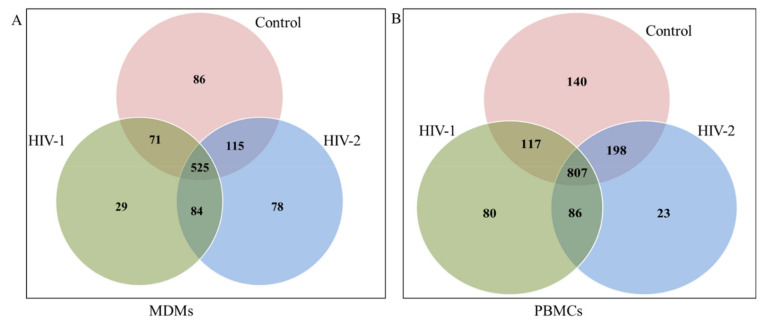
Total number of miRNAs overlapping between uninfected controls, HIV-1- and HIV-2-infected MDMs (**A**) and PBMCs (**B**).

**Figure 2:**
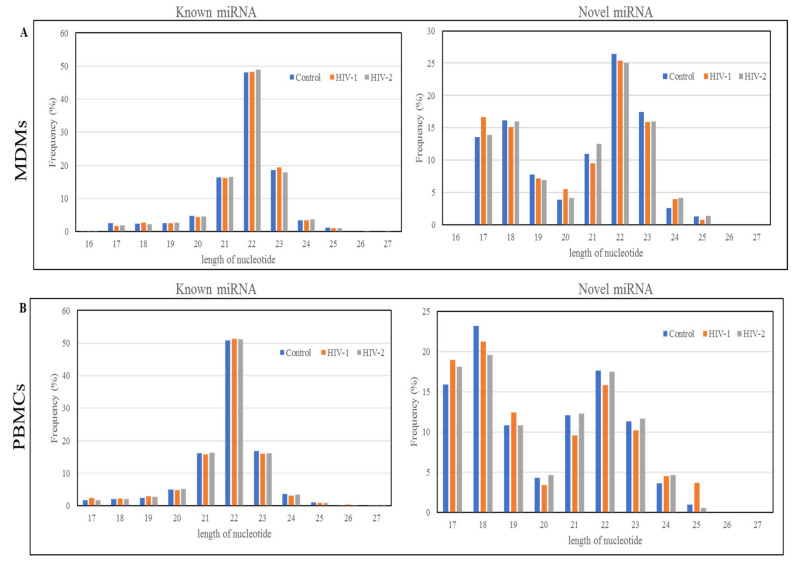
The length distribution and frequency of all the mapped miRNAs in MDMs (**A**) and PBMCs (**B**).

MiRNA sequencing has the ability to find novel as well as known miRNAs. In this study, the miRDeep software package [47] was used to detect known miRNAs, as well as to identify novel miRNAs. The secondary structure of novel miRNAs was predicted by the Randfold software. The results indicated that there were, in total, 990 miRNAs detected including 780 known-miRNAs and 210 predicted novel miRNAs in the control, HIV-1-infected, and HIV-2-infected MDMs. In PBMCs, 1450 miRNAs were detected in the three groups (control, HIV-1-infected, and HIV-2-infected PBMCs), including 918 known miRNAs and 532 novel predicted miRNAs (Table 3). The results of the Venn diagram (Figure 1A) indicate that a total of 525 miRNAs were common among all the three groups (control, HIV-1-infected, and HIV-2-infected MDMs), while 86 miRNAs were unique to uninfected control MDMs, 129 miRNAs were unique to HIV-1-infected MDMs, and 158 miRNAs were unique to HIV-2-infected MDMs, respectively. Similarly, in PBMCs, 807 miRNAs were commonly expressed among all three groups, while 140 miRNAs were unique to uninfected control PBMCs, 80 miRNAs were unique to HIV-1-infected PBMCs, and 23 miRNAs were unique to HIV-2-infected PBMCs, respectively (Figure 1B). The results of the length distribution of the mapped miRNAs indicated that the majority of the mapped known miRNAs were 20 to 24 nucleotides in length in MDMs and PBMCs, comprising 91.27~91.79% and 91.04~92.22% of their total number, respectively (Figure 2). The size distribution of novel miRNAs in MDMs and PBMCs were more heterogeneous. The majority of novel miRNAs in the MDMs were 22 nucleotides long, followed by 23, 18, and 17 nucleotides. In PBMCs, the majority of novel miRNAs were 18 nucleotides long, followed by 17, 22, 21, and 19 nucleotides (Figure 2).

### 2.3. Differentially Expressed miRNA between HIV-1- and HIV-2-Infected MDMs and PBMCs

**Figure 3:**
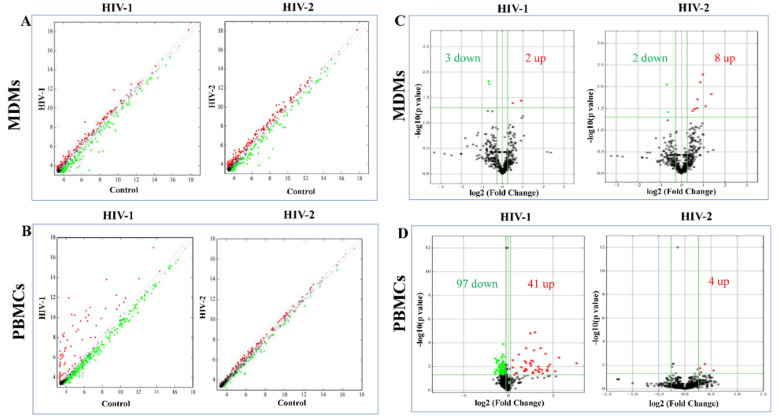
Scatter plots and volcano plots to identify differentially expressed miRNAs in MDMs and PBMCs. Scatter plots used to identify differentially expressed miRNAs in (**A**) HIV-1/HIV-2-infected MDMs versus uninfected control MDMs. (**B**) HIV-/HIV-2-infected PBMCs versus uninfected control PBMCs. The axis represents the mean normalized miRNA signal values for each comparator group (log2 scaled). The gray fold change lines represent 1.2× fold change. Red dots represent miRNAs that are upregulated (>1.2 fold) and green dots represents miRNAs that are downregulated (<1.2-fold). Volcano plots used to identify differentially expressed miRNAs in (**C**) HIV-1/HIV-2-infected MDMs versus uninfected control MDMs. (**D**) HIV-/HIV-2-infected PBMCs versus uninfected control PBMCs. The *x*-axis represents fold change values (log2 scale), while the *y*-axis represents *p*-values (−log10 scale). The green vertical lines correspond to 1.2× upregulation and downregulation, respectively, while the green horizontal line corresponds to a *p*-value of 0.05. The red dots represent differentially upregulated miRNAs and green dots represent downregulated miRNAs of statistical significance.

MiRNA expression levels were compared between the HIV-1- or HIV-2-infected groups in relation to the uninfected control group in order to identify differentially expressed miRNAs. For miRNAs expressed at very low levels, we added 10 units to each miRNA value to calculate the fold change. The changing trends of miRNA expression level were demonstrated by scatter plot (Figure 3A,B). Based on the criteria of a fold change of ≥1.2 for upregulated miRNAs, a fold change of ≤0.83 for downregulated miRNAs, and *p*-value ≤ 0.05, a total of 138 differentially expressed miRNAs were identified, with 41 miRNAs being upregulated and 97 miRNAs downregulated, between the HIV-1-infected PBMCs and control group (Figure 3D, Table S1.1). Among them, 96 were known miRNAs and 42 were predicted novel miRNAs. It is worth noting that hsa-miR-3195, hsa-miR-3656, hsa-miR-4492, and hsa-miR-6087 were detected only in the HIV-1-infected PBMC group and were not detected in the uninfected control group. This suggests that hsa-miR-3195, hsa-miR-3656, hsa-miR-4492, and hsa-miR-6087 could be sensitive biomarkers of HIV-1 infection in PBMCs after additional validation in patient samples. Moreover, in comparison to the uninfected control PBMCs, many of the known miRNAs, including hsa-miR-1273h-3p, hsa-miR-1273h-5p, hsa-miR-671-5p, and hsa-miR-7-5p were significantly downregulated. The expression of hsa-miR-novel-chr12_10302, hsa-miR-novel-chr3_50649, etc., were also significantly downregulated in the HIV-1-infected PBMCs (Table S1.1). In response to HIV-2 infection in PBMCs, only four miRNAs were upregulated in comparison to the uninfected control PBMC group (Figure 3D, Table S1.2). None of the miRNAs were significantly downregulated in HIV-2-infected PBMCs compared to uninfected control PBMCs. Among the upregulated miRNAs, two known miRNAs (hsa-miR-18a-3p and hsa-miR-320b) were only detected in HIV-2-infected PBMCs, making them promising biomarker candidates for detecting HIV-2 infection in PBMCs (Table S1.2). In order to investigate the differential expression of miRNAs in MDMs infected with HIV-1 or HIV-2 infection, selection criteria of fold change (cut-off value of ≥1.2 for upregulated miRNAs and a cut-off value of ≤0.83 for downregulated miRNAs) and *p*-value ≤ 0.05 were used. A total of 5 and 10 deregulated miRNAs were discriminated by this approach for HIV-1 and HIV-2, respectively (Figure 3C, Table S2). Interestingly, the majority of miRNAs were downregulated in HIV-1-infected MDMs; on the other hand, the majority of miRNAs were upregulated in HIV-2-infected MDMs compared to the uninfected control MDMs. Three miRNAs (hsa-miR-148b-5p, hsa-miR-26a-2-3p, and hsa-miR-novel-chr17_21688) were deregulated in HIV-1- and HIV-2-infected MDMs. (Figure 3C, Tables S2.1 and S2.2). Based on our results, hsa-miR-542-3p, hsa-miR-375, hsa-miR-195-5p, hsa-miR-30c-2-3p, hsa-miR-4802-3p, and hsa-miR-26b-5p exhibited significant upregulation in HIV-2-infected MDMs only, whereas hsa-miR-199a-1, miR-199a-2, and hsa-miR-874-5p displayed the most significant reduction in the HIV-1- and HIV-2-infected MDMs, respectively, compared to uninfected control MDMs (Table S2).

**Figure 4:**
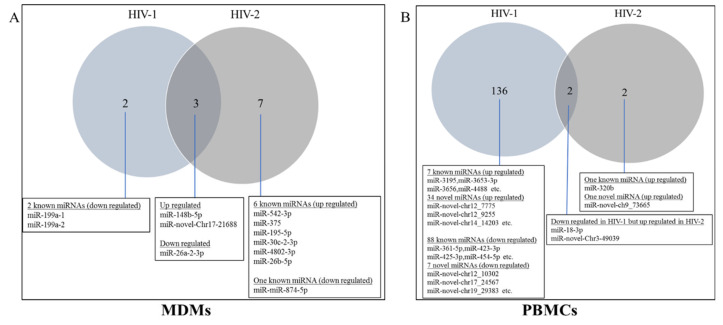
Analysis of common and unique differentially expressed miRNAs in HIV-1- and HIV-2-infected MDMs and PBMCs. Venn diagrams indicate the number of overlapping and non-overlapping differentially regulated miRNAs in MDMs (**A**) and PBMCs (**B**) infected with HIV-1 and HIV-2 compared to uninfected control MDMs and PBMCs.

In order to investigate the differences in molecular mechanisms related to replication capacity and infectivity, we compared the differentially expressed miRNAs of uninfected control vs. HIV-1 and uninfected control vs. HIV-2. The data indicated that only two differentially expressed miRNAs were common between HIV-1- and HIV-2-infected PBMCs. In PBMCs, 136 differentially expressed miRNAs were specific to HIV-1 infection and 2 differentially expressed miRNAs were specific to HIV-2 infection (Figure 4B). In MDMs, three differentially expressed miRNAs were common between HIV-1 and HIV-2 infection, two differentially expressed miRNAs were specific for HIV-1 infection, and seven differentially expressed miRNAs were specific only for HIV-2 infection (Figure 4A). Hsa-miR-148b-5p, hsa-miR-novel-chr17_21688, and hsa-miR-26a-2-3p were both deregulated in HIV-1- and HIV-2-infected MDMs (Figure 4A, Tables S2.1 and S2.2). Surprisingly, hsa-miR-18a-3p and hsa-miR-novel-chr3_49039 were significantly downregulated in HIV-1-infected PBMCs but were significantly upregulated in HIV-2-infected PBMCs (Figure 4B, Tables S1.1 and S1.2), suggesting that they play different roles in the regulatory mechanisms of infectivity and replication in HIV-1 and HIV-2 infection.

When differentially expressed miRNAs of the HIV-1- and HIV-2-infected groups were compared, 4 miRNAs were commonly regulated in both the HIV-1 and HIV-2 infection, whereas 138 differentially expressed miRNAs and 9 differentially expressed miRNAs were uniquely identified in the HIV-1 and HIV-2 samples compared with uninfected control samples (Table S3). These results suggest that different miRNAs exert different molecular functions with respect to the HIV-1 and HIV-2 responses in PBMCs and MDMs.

### 2.4. Validation of miRNA Expression by qRT-PCR

**Figure 5:**
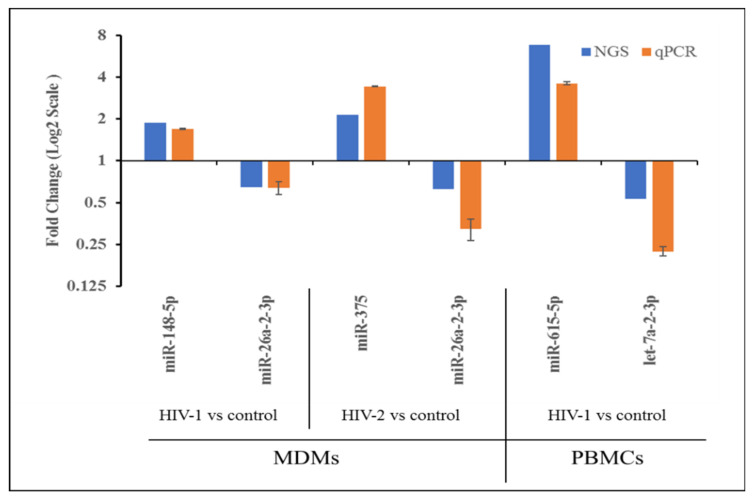
Expression pattern validation of miRNAs selected from the NGS data by qPCR.

To verify the results of the NGS and comparative analyses, we randomly selected 6 miRNAs for qPCR confirmation: one upregulated and one downregulated from HIV-1- and HIV-2-infected MDMs and PBMCs. Since, no downregulated miRNAs were identified in HIV-2-infected PBMCs, no miRNA was selected from HIV-2-infected PBMCs for validation. The qPCR detection of the differentially expressed miRNAs were consistent with those identified through NGS data, confirming the reliability of our differentially expressed miRNAs data (Figure 5).

The expression of selected miRNAs was tested in all the independent donor samples using real-time PCR (RT-PCR). The two-step RT-PCR method was used with a TaqMan MicroRNA Reverse Transcription Kit and TaqMan miRNA-specific primers (Life Technologies, Inc., Waltham, MA, USA) according to the manufacturer’s protocols. RNU44 was used as an internal control for normalization between each donor. Each sample was run in triplicate to ensure accurate fold change estimation. Relative miRNA expression was calculated by the 2^ ^[−{ΔCt (infected) − ΔCt (uninfected)}]^ formula. The results of the qPCR were analyzed in triplicate. Results expressed as mean ± SEM are representative of three independent donors.

### 2.5. Functional Annotation and KEGG Pathway Enrichment Analysis of Differentially Expressed miRNA Target Genes

To better understand the roles of the differentially expressed miRNAs identified in HIV-1- and HIV-2-infected MDMs and PBMCs, the target genes of these miRNAs were predicted as described in the materials and methods section. Target genes that overlapped with each database were selected for analysis. In HIV-1-infected PBMCs, 1631 common predicted target genes were identified that were putative targets of the upregulated miRNAs and 1205 common predicted target genes were identified that were putative targets of the downregulated miRNAs, respectively (Figure S3B). In HIV-2-infected PBMCs, a total of 155 putative target genes were identified by the four upregulated miRNAs (Figure S3B). In MDMs infected with HIV-1, 59 common predicted target genes were identified that were putative targets of the upregulated miRNAs and 168 common predicted target genes were identified that were putative targets of the downregulated miRNAs (Figure S3A). Similarly, for HIV-2-infected MDMs, 828 target genes were detected that were targeted by upregulated miRNAs and 230 common predicted genes were detected for the downregulated miRNAs, respectively (Figure S3A). As expected, most miRNAs targeted hundreds of genes (Figure S4). These targets were also analyzed by GO and KEGG pathway annotation.

To date, the functions of the majority miRNAs have not been annotated. The functional prediction of miRNAs is largely based on the annotations of their interacting protein-coding genes. To a certain extent, GO term and KEGG pathway enrichment analyses of dysregulated mRNAs can reveal the biological roles of the differentially expressed miRNAs that were selected. The results of a GO term enrichment analysis in HIV-1-infected MDMs indicate that the principally enriched significant terms included metabolic processes, biosynthetic process, regulation of cellular protein, DNA and protein binding activity (Table S4.1 and Figure S5A). GO analysis revealed enriched significant regulation of cellular and metabolic processes, kinase activity, apoptosis process and system development, and catalytic activity, in the case of HIV-2-infected MDMs (Table S4.2 and Figure S5B). Over-represented GO classes of the predicted target gene of the differentially regulated miRNAs in HIV-1- and HIV-2-infected PBMCs were clustered (Table S5). The results provided in Figure S5C,D show the top 10 most significantly enriched GO terms in the BP, CC, and MF categories for the up and downregulated miRNAs predicted to target mRNAs in HIV-1 and HIV-2 infected PBMCs. Since, no downregulated miRNAs were identified in HIV-2-infected PBMCs, therefore, there were no enriched GO terms associated with the downregulated miRNA-targeted genes.

**Figure 6:**
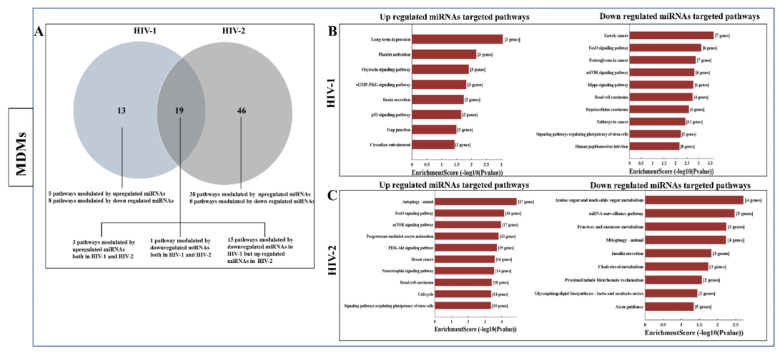

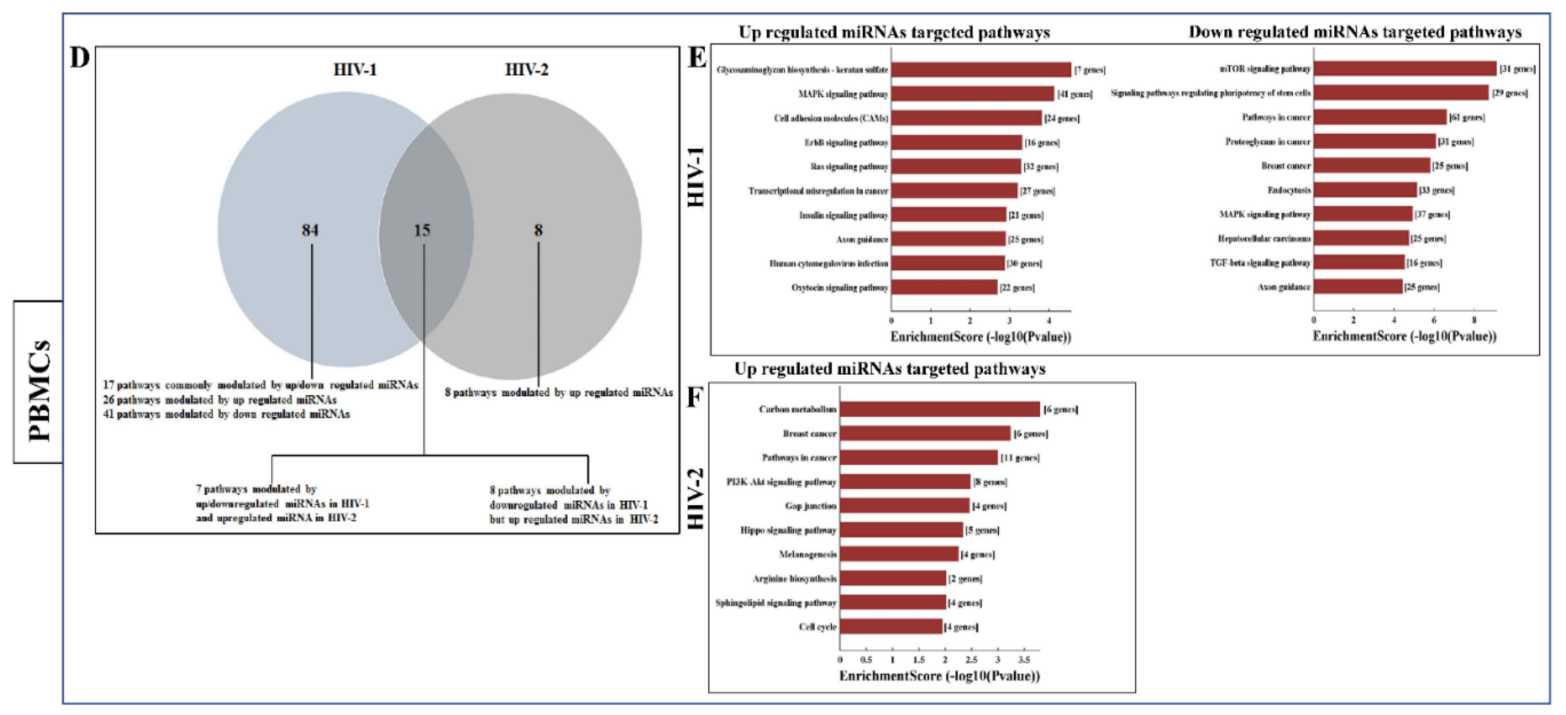
KEGG pathway analysis of differentially expressed miRNA-targeted genes in MDMs and PBMCs infected with HIV-1 and HIV-2: (**A**) Venn diagram indicates the numbers of overlapping and non-overlapping differentially regulated miRNA-targeted pathways in HIV-1 and HIV-2 infected MDMs. (**B**,**C**) The top ten KEGG pathways enriched in HIV-1 infected MDMs vs. uninfected controls (**B**) and HIV-2 infected MDMs vs. uninfected controls (**C**). The bar plot shows Fold Enrichment value of the significant enrichment terms and pathway analysis for differentially expressed upregulated and downregulated miRNA-targeted genes in HIV-1/HIV-2 infected MDMs, respectively. (**D**) Venn diagram indicates the numbers of overlapping and non-overlapping differentially regulated miRNA-targeted pathways in HIV-1 and HIV-2 infected PBMCs. (**E**,**F**) The top ten KEGG pathways enriched in HIV-1 infected PBMCs vs. uninfected controls (**E**) and HIV-2 infected PBMCs vs. uninfected controls (**F**). The bar plot shows Fold Enrichment value of the significant enrichment terms and pathway analysis for differentially expressed upregulated and downregulated miRNA-targeted genes in HIV-1/HIV-2 infected PBMCs, respectively.

Pathway analysis was performed using the KEGG database. The results indicated that 8 and 56 significant pathways were associated with mRNAs targeted by upregulated miRNAs in HIV-1 and HIV-2 infected MDMs (Tables S6.1 and S6.2), respectively. Remarkably, three of these pathways were shared between HIV-1 and HIV-2 infected MDMs (Figure 6A, Table S6). In contrast, 24 pathways for downregulated miRNA-targeted mRNAs were enriched only in the HIV-1 infected MDMs and 9 pathways for downregulated miRNA-targeted mRNAs were enriched only in HIV-2 infected MDMs (Tables S6.1 and S6.2). Only one pathway was enriched for downregulated miRNA-targeted mRNAs in both HIV-1 and HIV-2 infected MDMs. We found 15 pathways that were enriched in downregulated miRNA-targeted genes in HIV-1 infected MDMs. Interestingly, these same 15 pathways were found in upregulated miRNA-targeted genes in HIV-2 infected MDMs (Figure 6A). KEGG pathway analysis revealed that ‘long-term depression’, ‘platelet activation’, ‘renin secretion’, ‘gap junction’, and ‘circadian entrainment’ were upregulated only in HIV-1 infected MDMs (Figure 6B and Table S6.1); the downregulated miRNA-targeted mRNAs were mainly involved in ‘basal cell carcinoma’, ‘human papillomavirus infection’, ‘wnt signaling pathway’, and ‘melanogenesis’ (Figure 6B and Table S6.1). Genes involved in ‘autophagy’, ‘progesterone-mediated oocyte maturation’, ‘PI3K-Akt signaling pathway’, ‘neurotrophin signaling pathway’, ‘cell cycle’, and ‘Fc gamma R-mediated phagocytosis’ were found to be upregulated in HIV-2-infected MDMs (Figure 6C and Table S6.2). The downregulated miRNA-targeted mRNAs in HIV-2-infected MDMs were mostly related to ‘amino sugar and nucleotide sugar metabolism’, ‘mRNA surveillance pathway’, ‘fructose and mannose metabolism’, and ‘mitophagy’ (Figure 6C and Table S6.2). KEGG pathway analysis was also used to reveal the biological pathways of the differentially regulated miRNAs targeted genes in HIV-1 and HIV-2 infected PBMCs. The results indicated that 50 significant pathways in HIV-1 infected PBMCs and 23 significant pathways in HIV-2 infected PBMCs were associated with upregulated miRNA-targeted mRNAs (Table S7). Remarkably, seven of these pathways were shared between HIV-1 and HIV-2 infected PBMCs. In contrast, 73 pathways for downregulated miRNA-targeted mRNAs were enriched in the HIV-1-infected PBMCs. No pathways were identified for downregulated miRNA-targeted mRNAs, as no downregulated miRNAs in HIV-2 infected PBMCs were detected. Seventeen pathways were enriched by the up and downregulated miRNA-targeted genes in HIV-1 infected PBMCs. It is interesting to note that eight pathways that were enriched in downregulated miRNA-targeted genes in HIV-1 infected PBMCs were enriched in upregulated miRNA-targeted genes in HIV-2-infected PBMCs (Figure 6D, Tables S7.1 and S7.2). KEGG pathway analysis revealed that the ‘glycosaminoglycan biosynthesis—keratan sulfate’, ‘cell adhesion molecules’, and ‘oxytocin signaling pathway’ were upregulated only in HIV-1 infected PBMCs (Figure 6E); likewise, in HIV-1 infected PBMCs, the downregulated miRNA-targeted mRNAs were mainly involved in ‘mTOR signaling pathway’, ‘signaling pathways regulating pluripotency of stem cells’, ‘endocytosis’, and ‘TGF-beta signaling pathway’ (Figure 6E). Genes involved in ‘carbon metabolism’, ‘gap junction’, ‘arginine biosynthesis’, and ‘citrate cycle’ were found to be targeted by upregulated miRNAs in HIV-2 infected PBMCs (Figure 6F).

## 3. Discussion

HIV-1 and HIV-2 are closely related retroviruses that share many similar traits like modes of transmission, viral replication, and pathogenesis. However, major differences exist between the clinical outcomes presented by the two viruses. Most people infected with HIV-2 do not progress to disease [48,49], even though the minority who do progress cannot be distinguished clinically from HIV-1-infected patients. HIV-1 is more pathogenic than HIV-2, with higher measurable levels of plasma viremia; however, the exact mechanisms contributing to these differences is not completely understood. 

The use of next-generation sequencing (NGS) technologies has facilitated the detection of low levels of expressed miRNAs, which has enabled the discovery of novel miRNAs, as well as the detection of differentially expressed miRNAs [50,51]. A single miRNA has the potential to target more than one pathway through hundreds of distinct mRNA molecules and one pathway can be regulated by multiple miRNAs [52], thus miRNAs are attractive candidates as regulators of multiple pathways. However, it has become gradually clear that not all miRNAs are equally significant. Specific miRNAs emerge as principal regulators that control major cell functions in various physiological and pathological settings [53]. Human immunodeficiency virus (HIV) has developed several strategies to disseminate within the human body using host cell machinery [9,10,11,12,13]. It is well known that T cells die rapidly in patients after HIV-1 infection. By contrast, MDMs can survive infection and can migrate into all body tissues. MDMs are characteristically resistant to virus-mediated killing, functioning as an HIV-1 reservoir [14], and are thus suspected to be a major agent for spreading HIV [54]. Therefore, the identification of master miRNAs expressed in response to HIV-1 and HIV-2 infection in PBMCs and MDMs, the related pathways targeted by these miRNAs, and the underlying comparative molecular mechanisms exerted by these miRNAs are important areas of study in HIV-1 and HIV-2 research.

In the present study, we have investigated the differentially expressed miRNAs in HIV-1 or HIV-2 infected MDMs and PBMCs compared with uninfected cells. To our knowledge, this is the first study to profile the expressed miRNAs and analyze their targeted pathways in HIV-1 and HIV-2 infected MDMs and PBMCs side by side using NGS. We have previously evaluated [47] the differentially expression of miRNAs in PBMCs infected with HIV-1 and HIV-2 using PCR-arrays. The use of PCR arrays in the previous study enabled us to evaluate only a small number of miRNAs. Using NGS too in the current study helps us to evaluate the comparative global miRNA expression profile in MDMs and PBMCs infected with HIV-1 and HIV-2. The integration of NGS technology, differentially expressed miRNA analysis, target prediction, and functional annotation analyses of targets has allowed us to perform a robust comparative genomics and bioinformatics study to reveal host miRNA molecular signatures associated with HIV-1 and HIV-2 infection. It is known that macrophages contribute to the persistence and pathogenesis of HIV. Whereas HIV-infected CD4+ T cells die within a few days of infection, in vitro studies suggest that macrophages are resistant to the cytopathic effects of HIV replication. Here, we have characterized and compared the interactions of miRNAs specific to targeted pathways in HIV-1-infected MDMs and PBMCs, to understand the probable cause of viral persistence in the two cellular compartments. Our results identified cellular pathways associated with the differentially expressed host miRNAs during the HIV-1 and HIV-2 life cycles in MDMs and PBMCs. We identified a unique series of cellular miRNAs, providing, for the first time, key molecular insights into unique cellular miRNA target interactome networks dynamically and temporarily affected by HIV-1 and HIV-2 infection.

As shown in Figure 3C,D (see also Tables S1.1 and S2.1), compared with the uninfected control, there are 5 differentially expressed miRNAs in HIV-1 infected MDMs and 138 differentially expressed miRNAs in HIV-1 infected PBMCs. Most of the differentially expressed miRNAs are downregulated in both HIV-1 infected MDMs and PBMCs. Taken together, these results suggest that a large number of miRNAs are downregulated, resulting in the upregulation of their mRNA targets in HIV-1 infected cells. In HIV-2 infected PBMCs, no statistically significant miRNAs were found to be downregulated. However, in MDMs, 80% of the differentially expressed miRNAs were upregulated (Figure 3C,D, Tables S1.2 and S2.2). Among these differentially expressed miRNAs, consistent with previous studies, many miRNAs or their homologs were reported to be closely related with HIV infection. For instance, miR-18a-3p, miR-23b-5p, and miR-25-5p were differentially expressed in HIV-1 infected PBMCs [55]; let-76-5p, let-7g-5p, and miR-16-2 were differentially expressed in HIV-1 infected patient plasma [56]; miR-148b-5p, miR-26a-2-3p, miR-23b-5p, miR-25-5p, and mi-939-5p were differentially expressed in PBMCs infected with HIV-2 virus [55]. However, there are still some differences between our results and previous studies [55,57]. We have found that miRNA expression in HIV-1 and HIV-2 infected PBMCs was 62.5% and 50%, respectively, concordant with our previously published papers (Table S8) [55]. We speculate that these differences may be attributed to the different type of analysis methods and host to host variations. In our previous study, we have only reported miRNA expression profiles in samples and did not consider donor to donor variations in miRNA expression. However, in our current study, we have used *p*-value as an additional criterion to select the differentially expressed miRNA. The *p*-value suggests how much the expression value of a particular miRNA in the infected sample varies and therefore, it is a measure of how affected the miRNA was by the infection. Thus, by using a more stringent criterion for selection, we have found fewer differentially expressed miRNAs in our current study. We have also identified many interesting differentially expressed miRNAs that so far have not been associated with HIV infection but have been linked to other diseases [58]. For example, miR-326, miR-185, miR-191, and miR-425 were reported to play an important role in cancer and in the regulation of tumorigenesis [59,60,61,62,63,64,65]. 

Of these differentially expressed miRNAs, it appears miR-26a-2-3p might be a specific biomarker of HIV-1 infection, since its expression was decreased by 0.6-fold over the control uninfected group (Tables S1.1 and S2.1) in both HIV-1-infected PBMCs and MDMs. We also observed (Figure 4B) that miR-18-3p was downregulated in HIV-1-infected PBMCs, while being upregulated in HIV-2-infected PBMCs, suggesting that miR-18-3p may play different roles in the regulation of HIV-1 and HIV-2 infection in PBMCs. Reynoso et al. reported that miR-18a-3p was downregulated in chronic HIV-1-infected plasma samples [66]. Reports suggest that miR-18a-3p regulates autophagy pathways [67], directly targets the 3′UTR of the ataxia telangiectasia mutated (ATM) gene, and regulates DNA damaging activity in Epstein–Barr virus-associated lymphomas [68]. The ATM protein is involved in enhancing HIV-1 replication by stimulating Rev function [69]. In fact, ATM kinase is an important transducer of the DNA damage signal [68]. In contrast, miR-18a-3p is upregulated in HIV-2-infected PBMCs leading to the downregulation of mRNA targets, which may help abrogate the cell cycle arrest triggering apoptosis, as found in cancer cells [70,71]. Thus, our results suggest that the differential expression of miR-18a-3p may contribute to the significant differences observed in the autophagy and apoptosis pathways in HIV-1 and HIV-2 infections.

Kyoto Encyclopedia of Genes and Genomes (KEGG) pathway analysis, comparing patterns of pathway activation in HIV-1 and HIV-2 infection in MDMs, showed that several cellular pathways had dissimilar levels of activation (Figure 6A–C). For instance, FoxO signaling pathways were modulated by downregulated miRNAs in HIV-1-infected MDMs (Table S6.1) and upregulated miRNAs in HIV-2-infected MDMs (Table S6.2). FOXO proteins play an essential role in the crosstalk between many signaling pathways, including cell cycle, metabolism, apoptosis, and cell survival [72,73,74]. Regulation of FOXO transcription factors is carried out by a complex interplay of phosphorylation, acetylation, ubiquitination, and interaction with other protein partners [75,76,77,78,79,80]. It is speculated that with the downregulation of miRNAs, FOXO signaling pathway-related proteins are upregulated in HIV-1-infected MDMs, and may contribute to HIV-1-mediated cell death of macrophages during productive infection [81]. 

Many studies indicate that inflammatory responses might result in HIV-induced pathogenesis. Specially, one of the major aspects of CD4+ T cell depletion and its associated immunopathology that distinguishes between HIV-1 and HIV-2 infections is immune activation [82], which is a strong predictor of disease progression in HIV infection [83]. Earlier studies have revealed that in HIV-1 infection, as disease progresses, the frequency of CD4+ T cells is found to decline [84], which in turn relates to the reduced renewal capacity and increased susceptibility of these populations of cells to apoptosis [85,86]. However, in HIV-2 infection, the proportion of CD4+ T cells is well preserved [84]. Interestingly, in HIV-1-infected PBMCs, there are several downregulated miRNAs whose targets are genes that regulate the Hypoxia-inducible factor 1 (HIF-1) signaling pathway (Table S7.1). Hypoxia-inducible factor 1α (HIF-1α) is a transcriptional activator factor that plays a central role in the HIF-signaling pathway. After HIF-1α is translocated to the nucleus, it binds to the hypoxia-responsive element (HRE) which is present in the promoters of numerous apoptosis pathway-related genes like p53, Bax, and Bak that are upregulated leading to CD4+ T cells depletion. On the other hand, in HIV-2-infected PBMCs, the HIF-1 signaling pathway is modulated by upregulated miRNAs (Table S7.2) that serve as negative regulators for HIF-1 signaling and hypoxia-mediated apoptosis during HIV-2 infection, preventing the depletion of CD4+ T cells. We observed that 24 pathways were modulated by both up and downregulated miRNAs in HIV-1-infected PBMCs (Figure 6D and Table S7.1). It is therefore intriguing to speculate that during the infection of PBMCs with HIV-1, the virus turns on many signaling pathways to favor their replication; however, to defend against virus infection in the host, most of sthe ignaling pathways are turned off by the host to avoid severe tissue damage.

In HIV-1 infection, we have found that of the 154 pathways examined (Tables S6.1 and S7.1), only 24 pathways were commonly modulated in MDMs and PBMCs (Table 4). This signifies that HIV-1 pathogenesis in the two different host cells, MDMs and PBMCs, acts in different ways. A comparison of patterns of pathway activation in MDMs and PBMCs during HIV-1 infection showed that several cellular pathways had dissimilar levels of activation. The p53 signaling pathway is heavily involved in determination of the fate of a cell and has been implicated in mediating cell cycle arrest during viral infection [87]. The tumor suppressor p53 plays a key role in the stimulation of cell apoptosis and growth arrest through a cooperative signaling network of genotoxic stress caused by treatment with anticancer drugs, irradiation, ultraviolet light, and glucose starvation [88]. HIV-1 infection also induces genotoxic stresses linked to p53 activation in CD4+ T cells by integration-mediated dsDNA strands break, secretion of type I interferons, and expression of HIV-1 accessory proteins Vpr/Vpu, which may be considered as intracellular markers of HIV-1 infection [89,90,91,92]. p53 activation by HIV-1 infection also plays an important role in host-restriction mechanisms against HIV-1 replication, which suppress long terminal repeat (LTR)-mediated viral transcription through Tat modulation [93]. These findings directly correlate with our observations indicating that in PBMCs, downregulated miRNAs canonically activate p53 signaling pathways, through upregulation of p53 pathway-related genes. Breton et.al have reported inhibition of p53 signaling in macrophages during the early post entry steps of HIV-1 infection [94]. Likewise, in HIV-1-infected MDMs, our results indicate that p53 signaling pathway-related genes are downregulated by the upregulated miRNAs. This may be one reason why macrophages are resistant to HIV-1-mediated cytopathic effects and serve as viral reservoirs, contributing to virus persistence in an infected individual. 

Comparing patterns of pathway modulation in PBMCs and MDMs during HIV-2 infection showed that several cellular pathways had dissimilar levels of modulation, but only 13 pathways were commonly modulated (Table 5). This signifies that HIV-2 pathogenesis in PBMCs and MDMs acts in different ways. Among common pathways, the PI3K-Akt signaling pathway was modulated by upregulated miRNAs in both the cell types during HIV-2 infection. This indicates that PI3K-Akt signaling pathway-related genes are downregulated during HIV-2 infection. On the contrary, reports indicate that PI3K-Akt signaling pathway-related genes are activated in both T cells [95] and macrophages [96] during HIV-1 activation. The delayed disease progression observed in HIV-2-infected individuals could be due to the differences in the activation state of signaling pathway-related genes like the PI3K-Akt signaling pathway. It is important to note that our findings are based on in silico studies and need to be further validated. 

In summary, using state-of-the-art NGS data analysis, comprising read counting, normalization, and calculations of fold changes, we investigated a total of 990 miRNAs in MDMs and 1450 miRNAs PBMCs, respectively, overlapping them with the miRBase 21 miRNAs data base. We identified 780 known miRNAs and 210 predicted novel miRNAs in MDMs, and 918 known miRNAs and 532 novel predicted miRNAs in PBMCs. For each differentially expressed miRNA, we investigated the following aspects: (1) miRNA synonyms, (2) functional information, (3) different isoforms, (4) novel miRNA detection, (5) description of novel miRNAs, (6) expression profile changes, (7) validation of selected miRNAs, (8) conserved targets of known miRNAs using mirdvV5 and TargetScan7.1, and (9) functional annotations of miRNA target genes using GO and KEGG database. The result of this multidimensional bioinformatics analysis is a comprehensive supplement that provides quick insights into how individual miRNAs of interest are regulated during HIV-1 and HIV-2 infections via the modulation of targeted pathways. The data collected and presented here serve as a valuable source of information for generating and testing hypotheses concerning the regulatory circuits that are active in HIV infected cells. In conclusion, here, we report a systematic and comprehensive computational study which provides a basis for further research into the pathogenesis of HIV. The study findings contribute significantly to our understanding of the molecular basis for the differences in clinical outcomes of HIV-1 and HIV-2 infection.

## 4. Materials and Methods

### 4.1. Peripheral Blood Mononuclear Cells (PBMCs) and Monocyte-Derived Macrophages (MDMs) Cell Culture

Human buffy coats and monocytes from individual donors seronegative for both HIV-1 and hepatitis B were provided by the National Institute of Health (NIH) blood bank. The NIH ethics committee approved this protocol to use deidentified samples of blood and/or blood products originally obtained under the NIH IRB-approved protocol and consent form (study number: 99-CC-0168, PI: Susan F. Leitman, M.D. dated on 04/09/12). Prior to initiation, this study protocol was approved by the FDA IRB. The PBMC isolation procedure from buffy coats was described earlier [55]. Briefly, monocytes (>95%) were removed by adherence to the culture flasks and the remaining PBMCs were stimulated for 72 h by culturing in RPMI-1640 medium supplemented with 10% heat inactivated FBS, penicillin-streptomycin, 10 U/mL IL-2, and 10 μg/mL of PHA. The PBMC were cultured for 24 h post stimulation in regular media prior to infection. 

Monocytes were isolated from PBMC of donors seronegative for HIV-1 and hepatitis B after leukapheresis and purified by countercurrent centrifugal elutriation as described earlier [29]. Cell suspensions contained >95% monocytes by the criteria of cell morphology on Wright-stained cytosmears, by granular peroxidase, and by non-specific esterase. The monocytes were differentiated in Dulbecco’s Modified Eagle Medium (DMEM) (Quality Biologicals, Gaithersburg, MD, USA) supplemented with 10% FBS, 100 units/mL Penicillin and 100 μg/mL Streptomycin/mL for 5–7 days in the presence of 0.02 μg/mL macrophage colony stimulation factor (MCSF, Cat#PHC9504, Thermo Fisher, Fredrick, MD, USA) at a density of 10^6^ cells/mL. Cells were judged by morphological examination and it was found that more than 98% cells were differentiated to macrophages.

### 4.2. Virus Infection in MDMs and PBMCs

PBMC cells were infected with known amounts (5 ng/mL HIV-1 p24 units per 10^6^ cells) of T-tropic HIV-1 (MN) and adherent MDMs were infected with equivalent amounts of (5 ng/mL HIV-1 p24 units/10^6^ cells of M-tropic HIV-1 Ba-L, Cat# 510, NIH AIDS Reagent, Bethesda, MD, USA) viruses. Both the cells were infected with equivalent amounts of dual tropic HIV-2 (ROD, 5 ng/mL SIV p27 units/million cells, Cat# 0121, NIBSC AIDS Reagent Program). After two hours post-infection, unadsorbed viruses were removed by washing the cells with PBS and cultured in complete media until further use. The cells were harvested on day 7 post infection as indicated. HIV-1 and HIV-2 replication was quantitated by an Alliance HIV-1 p24 Antigen ELISA Kit (Perkin Elmer, Waltham, MA, USA) and a RETRO-TEK SIV p27 Antigen ELISA Kit (ZeptoMetrix, Buffalo, NY, USA).

### 4.3. Total RNA Isolation

Total RNA was extracted from all samples using the miRNeasy total RNA isolation kit (QIAGEN, Valencia, CA, USA) according to the manufacturer’s protocols. RNA purity was measured using the NanoDrop ND-1000 spectrophotometer (Thermo Scientific Inc, San Diego, CA, USA). RNA concentration and integrity were evaluated using the Agilent Bioanalyzer 2100 system (Agilent Technologies, Santa Clara, CA, USA).

### 4.4. Small RNA Library Preparation and Sequencing

Total RNAs of each sample for small RNA library preparation and deep sequencing (Arraystar Inc, Rockville, MD, USA) were analyzed. An Illumina TruSeq Small RNA Sample Preparation Kit was used to prepare total RNA specimens. The 3’-adapter ligation with T4 RNA ligase 2 (truncated); 5’-adapter ligation with T4 RNA ligase; cDNA synthesis with RT primer; PCR amplification; extraction and purification of ~130-150 bp PCR amplified fragments (correspond to ~15–35 nt small RNAs) from the 15% PAGE gel were carried out according to the manufacturer’s instructions. After the completed libraries were quantified and integrity evaluated using with Agilent 2100 Bioanalyzer, the DNA fragments in the libraries were denatured with 0.1 M NaOH to generate single-stranded DNA molecules, captured on Illumina flow cells, amplified in situ, and finally, sequenced for 51 cycles on Illumina HiSeq according to the manufacturer’s instructions.

### 4.5. Initial Processing of Reads

Raw sequences were generated as clean reads from Illumina HiSeq by real-time base calling and quality filtering. The clean reads were recorded in FASTQ format, containing the read information, sequences, and quality encoding. Subsequently, the 3′ adapter sequence was trimmed from the clean reads and the reads with lengths shorter than 15 nucleotides (nt) were discarded. As the 5′-adaptor was also used as the sequencing primer site, the 5′-adaptor sequence was not present in the sequencing reads. The trimmed reads were recorded in FASTA format. For miRNA alignment, the maximum mismatch was 1. Reads with counts less than 2 were discarded when calculating miRNA expression. The alignment results were saved in text files. Only perfectly matched sequences could be set aside. First, the small RNA sequences were annotated with tRNA, rRNA, snRNA, snoRNA, and piRNA from NCBI GenBank and Rfam 9.1 databases, and discarded the matched ones. To avoid repeat associated sequences, reads with more than five total matching positions in the genome were removed. The repeat overlapping sequences were annotated as repeat associated small RNAs, and the sequences overlapping with the predicted exons and introns were filtered. More than 15 nt trimmed reads were aligned to the human pre-miRNA in miRBase 21, using novoalign software. Small RNA sequences that could not be matched to any category were used to predict novel miRNAs. The total numbers of the reads at the sequencing data processing stages are listed for each sample in Table 2.

### 4.6. Normalization of miRNA Expression

To compare the estimated miRNA expression, the miRNA expression levels were normalized, since the total number of reads from different samples may not be the same, and different sequencing depths may result in variations in the number of clones of individual miRNA. The miRNA expression levels were measured and normalized as transcripts per million of total aligned miRNA reads (TPM).

### 4.7. Known miRNA Expression Analysis

Normalized miRNA read counts were used to estimate the expression level of each miRNA. MiRNA expression differences were calculated between infected versus uninfected control groups and the “fold change” (i.e., the ratio of the group averages) and *p*-value between each group were computed. MiRNAs having fold changes ≥1.2 for upregulated miRNAs, a fold change of ≤0.83 for downregulated miRNAs, and *p*-value ≤ 0.05 were selected as the differentially expressed miRNAs. 

### 4.8. Novel miRNA Prediction

Novel miRNAs were predicted by algorithms such as miRDeep [47]. By using a simple model for miRNA precursor processing by Dicer, miRDeep is capable of both recovering the majority of known miRNAs present in heterogeneous deep-sequencing samples and reporting novel miRNAs with high confidence.

### 4.9. Validation of Mature miRNA Expression by qPCR

Validation of miRNA expression was performed by real-time PCR (RT-PCR) experiments as described earlier [55]. Briefly, cDNA was synthesized with a TaqMan miRNA reverse transcription kit according to the manufacturer’s instructions (Applied Biosystems, Pleasanton, CA, USA). RT-qPCR was performed in triplicate using 15 mM dNTPs, MultiScribe™ Reverse Transcriptase 50 U, 1.5 µL reverse transcription buffer, RNase inhibitor 3.8 U, and 3 µL of 5× RT primers for miRNA specific. qRT-PCRs were run on the ViiA7 fast real-time PCR system following the manufacturer’s suggested cycling parameters (Life Technologies, Carlsbad, CA, USA). The results were expressed as fold difference in expression of miRNA of interest relative to endogenous controls (RNU44) for infected and uninfected samples.

### 4.10. MiRNA Target Prediction, Gene Ontology, and Pathway Analysis

The conserved targets of known miRNA candidates were predicted using two databases (mirdvV5, and TargetScan7.1). Functional annotations of miRNA target genes were performed by the following databases: KO (KEGG Ortholog database) and GO (Gene Ontology). GO analysis helps to associate each targeted gene of differentially expressed miRNA with its function based on GO categories and facilitates the understanding of the intricate network of the targeted gene and its impact on viral replication. KEGG pathway was used for functional analysis mapping of the miRNA-targeted genes. The *p*-value (EASE-score, Fisher’s *p*-value, or Hypergeometric *p*-value) denotes the significance of the pathway correlated to the conditions. The lower the *p*-value, the more significant the pathway is (the recommended *p*-value cut-off is 0.05.)

## Figures and Tables

**Table 1:** Quantification of HIV-1 (P-24) and HIV-2 (P-27) antigens.

Sample Name	HIV-1 (pg/mL)	HIV-2 (pg/mL)
Cell Type: Peripheral Blood Mononuclear Cells (PBMCs)		
Donor 1	3148.13	28,011.78
Donor 2	4019.29	27,121.72
Donor 3	1653.06	24,534.10
Cell Type: Monocytes-Derived Macrophages (MDMs)		
Donor 1	1468.62	17,981.50
Donor 2	5451.53	22,746.26
Donor 3	2065.89	18,446.87

**Table 2:** Overview of the miRNA sequencing data in three independent PBMC donors and three independent MDM donors.

Sample Name	Clean Reads	Adapter-Trimmed Reads (Length ≥ 15 nt)	Reads Aligned to Human Pre-miRNA in miRBase 21
Cell Type: Peripheral Blood Mononuclear Cells (PBMCs)			
Control			
Donor 1	16,367,003	15,696,848	8,684,872
Donor 2	4,495,971	4,366,075	2,816,056
Donor 3	13,046,715	12,445,186	6,553,373
HIV-1			
Donor 1	17,138,982	15,436,055	6,409,199
Donor 2	20,145,570	18,802,590	8,531,490
Donor 3	11,429,935	10,390,651	5,447,327
HIV-2			
Donor 1	14,140,517	13,362,208	8,078,730
Donor 2	18,455,281	18,073,452	11,485,496
Donor 3	19,037,081	18,423,451	11,583,858
Cell Type: Monocytes-Derived Macrophages (MDMs)			
Control			
Donor 1	1,284,807	919,078	295,694
Donor 2	1,509,542	1,373,235	587,411
Donor 3	1,730,255	1,687,860	1,005,415
HIV-1			
Donor 1	1,246,998	949,827	466,372
Donor 2	1,378,466	1,166,657	386,670
Donor 3	1,782,736	1,706,479	1,008,880
HIV-2			
Donor 1	2,278,656	1,898,057	711,737
Donor 2	1,365,011	1,194,546	559,974
Donor 3	1,993,830	1,856,716	1,045,156

**Table 3:** Total number of known and novel miRNAs detected by next-generation sequencing in uninfected controls and HIV-1/HIV-2-infected PBMCs and MDMs.

Samples	Known-miRNAs	Novel-miRNAs	Total
Cell Type: Peripheral Blood Mononuclear Cells (PBMCs)			
Control	848	414	1262
HIV-1	737	353	1090
HIV-2	772	342	1114
Total	918	532	1450
Cell Type: Monocytes-Derived Macrophages (MDMs)			
Control	642	155	797
HIV-1	585	126	709
HIV-2	658	144	802
Total	780	210	990

**Table 4:** Common pathways modulated by differentially expressed miRNAs in HIV-1-infected MDMs and PBMCs.

Pathway Name	HIV-1					
	MDMs			PBMCs		
	Enrichment Score	*p*-Value	Pathways Modulated by Up/Downregulated miRNAs	Enrichment Score	*p*-Value	Pathways Modulated by Up/Downregulated miRNAs
Oxytocin signaling pathway	1.905063	0.01244335	up	2.692827	0.002028492	up
Renin secretion	1.734644	0.01842282	up	1.661208	0.02181686	up
p53 signaling pathway	1.651102	0.02233047	up	2.98958	0.001024284	down
mTOR signaling pathway	2.810829	0.001545864	down	9.093665	0.0000000008060005	down
Signaling pathways regulating pluripotency of stem cells	2.228881	0.005903624	down	8.711413	0.000000001943511	down
Pathways in cancer	2.421814	0.003786047	down	6.618831	0.0000002405298	down
Hepatocellular carcinoma	2.576053	0.002654281	down	4.732601	0.00001850968	down
Hippo signaling pathway	2.767124	0.001709528	down	4.424071	0.00003766423	down
FoxO signaling pathway	3.114274	0.0007686461	down	3.539176	0.0002889511	down
Gastric cancer	3.65153	0.0002230849	down	3.28796	0.0005152755	down
HTLV-I infection	1.71839	0.0191254	down	2.912919	0.001222027	down
Wnt signaling pathway	2.13995	0.0072452	down	2.590099	0.002569812	down
Chronic myeloid leukemia	1.595796	0.0253632	down	2.2901	0.00512743	down
ErbB signaling pathway	1.470936	0.03381145	down	3.314262	0.0004849954	up
				2.361913	0.004345974	down
Ras signaling pathway	1.358576	0.0437949	down	3.285602	0.0005180819	up
				3.212785	0.000612653	down
Human papillomavirus infection	2.165002	0.006839085	down	2.123619	0.007522818	up
				3.956626	0.000110503	down
Renal cell carcinoma	1.705678	0.01969345	down	1.890512	0.01286731	up
				4.353166	0.00004434387	down
Colorectal cancer	1.458046	0.03483006	down	1.586711	0.02589933	up
				3.290669	0.0005120717	down
Breast cancer	2.127675	0.007452897	down	1.586524	0.02591051	up
				5.795785	0.000001600352	down
Proteoglycans in cancer	2.872341	0.001341712	down	1.572002	0.02679158	up
				6.080734	0.00000083036	down
Melanogenesis	2.00586	0.009865974	down	1.423319	0.03772947	up
				1.777427	0.0166945	down
Cushing syndrome	2.044558	0.009024896	down	1.410542	0.03885604	up
				2.338646	0.004585155	down
Basal cell carcinoma	2.739614	0.001821318	down	1.358427	0.04381	up
				2.95548	0.001107949	down

**Table 5:** Common pathways modulated by differentially expressed miRNAs in HIV-2-infected MDMs and PBMCs.

Pathway Name	HIV-2					
	MDMs			PBMCs		
	Enrichment Score	*p*-Value	Pathways Modulated by Up/Downregulated miRNAs	Enrichment Score	*p*-Value	Pathways Modulated by Up/Downregulated miRNAs
PI3K-Akt signaling pathway	3.708008	0.0001958807	up	2.475812	0.003343395	up
Breast cancer	3.577508	0.0002645407	up	3.235154	0.0005818966	up
Cell cycle	3.358965	0.000437557	up	1.936352	0.01157838	up
Pathways in cancer	3.248617	0.0005641353	up	2.994257	0.001013313	up
Fc gamma R-mediated phagocytosis	2.996289	0.001008581	up	1.570297	0.02689695	up
Ras signaling pathway	2.981019	0.001044674	up	1.614705	0.02428257	up
Hippo signaling pathway	2.445519	0.003584934	up	2.333154	0.004643505	up
Endocrine resistance	2.229404	0.005896524	up	1.487751	0.03252735	up
Sphingolipid signaling pathway	1.691501	0.02034693	up	2.010001	0.009772351	up
Cushing syndrome	1.670783	0.02134112	up	1.623592	0.02379075	up
Hepatocellular carcinoma	1.415382	0.03842537	up	1.50253	0.03143911	up
Amino sugar and nucleotide sugar metabolism	2.710335	0.001948342	down	1.338931	0.04582143	up
Fructose and mannose metabolism	2.23792	0.005782019	down	1.639237	0.02294895	up

## Supplementary Materials

The following are available online at https://www.mdpi.com/1422-0067/21/18/6970/s1.

## Additional Information

This article reflects the views of the authors and should not be construed to represent FDA’s views or policies.
