# Supplementary material for: Comparison of miRNA Expression Profiles between HIV-1 and HIV-2 Infected Monocyte-Derived Macrophages (MDMs) and Peripheral Blood Mononuclear Cells (PBMCs)
Source: Int J Mol Sci. 2020 Sep 22;21(18):6970. doi: 10.3390/ijms21186970 (PMC7556008; doi:10.3390/ijms21186970)
Supplement: Supplementary file 1 [file ijms-21-06970-s001.zip › Figure S1 .pdf]

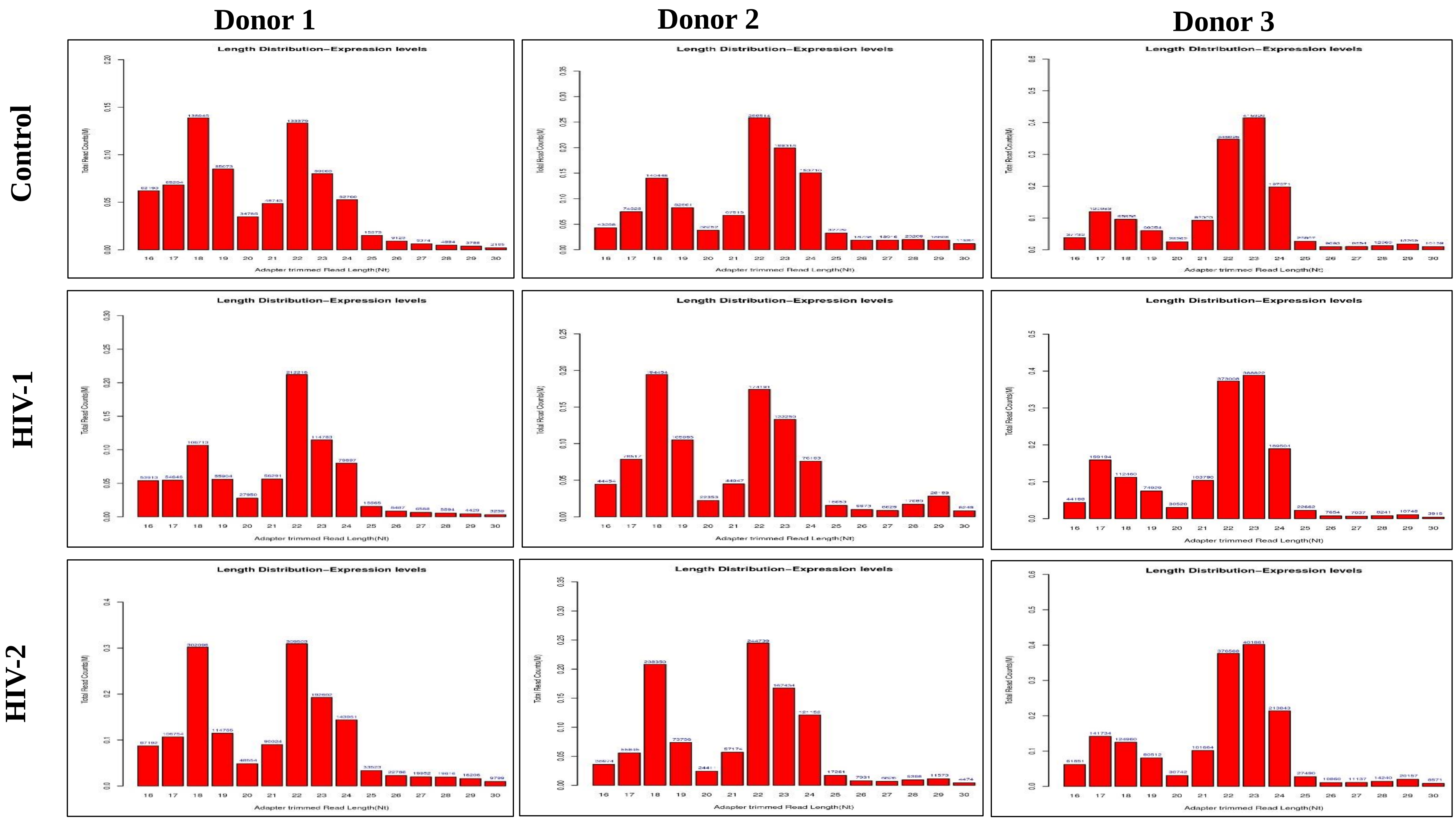
Donor 1
Donor 2
Donor 3
Control
HIV-1
HIV-2
Length Distribution–Expression levels
Total Read Counts(M)
Adapter trimmed Read Length(Nt)

(A) MDM

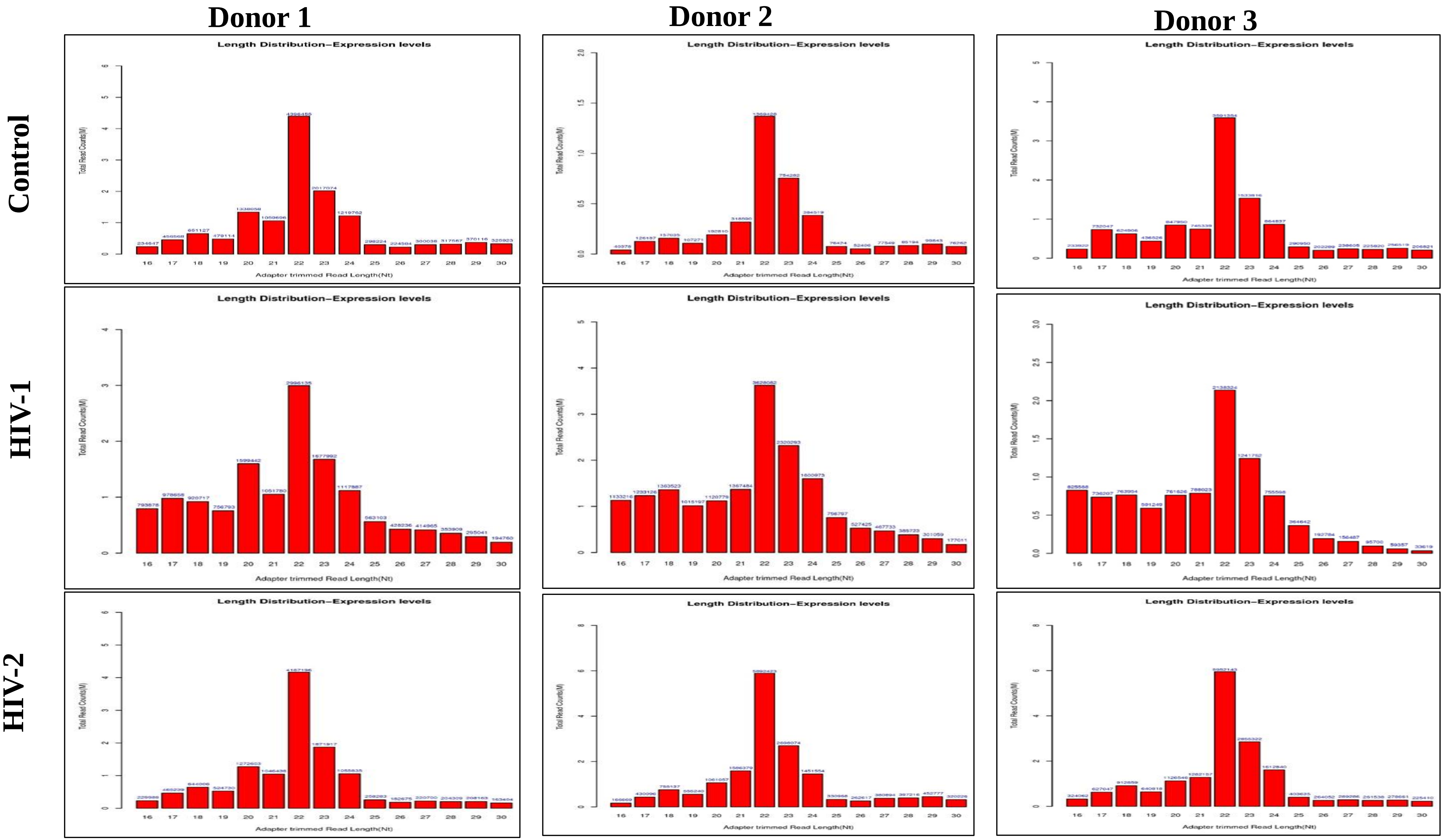
Donor 1
Donor 2
Donor 3
Control
HIV-1
HIV-2
Length Distribution−Expression levels
Total Read Counts(M)
Adapter trimmed Read Length(Nt)

(B) PBMC

Figure S1: **The length distribution of total small RNAs in all eighteen libraries.**

The distribution of small RNAs in MDMs (A) and PBMCs (B) for each group. The vertical axis indicates the total read counts and the horizontal axis indicates the read lengths for the complete adapter-trimmed read sets.
