# Supplementary material for: Comparison of miRNA Expression Profiles between HIV-1 and HIV-2 Infected Monocyte-Derived Macrophages (MDMs) and Peripheral Blood Mononuclear Cells (PBMCs)
Source: Int J Mol Sci. 2020 Sep 22;21(18):6970. doi: 10.3390/ijms21186970 (PMC7556008; doi:10.3390/ijms21186970)
Supplement: Supplementary file 1 [file ijms-21-06970-s001.zip › Figure S2 .pdf]

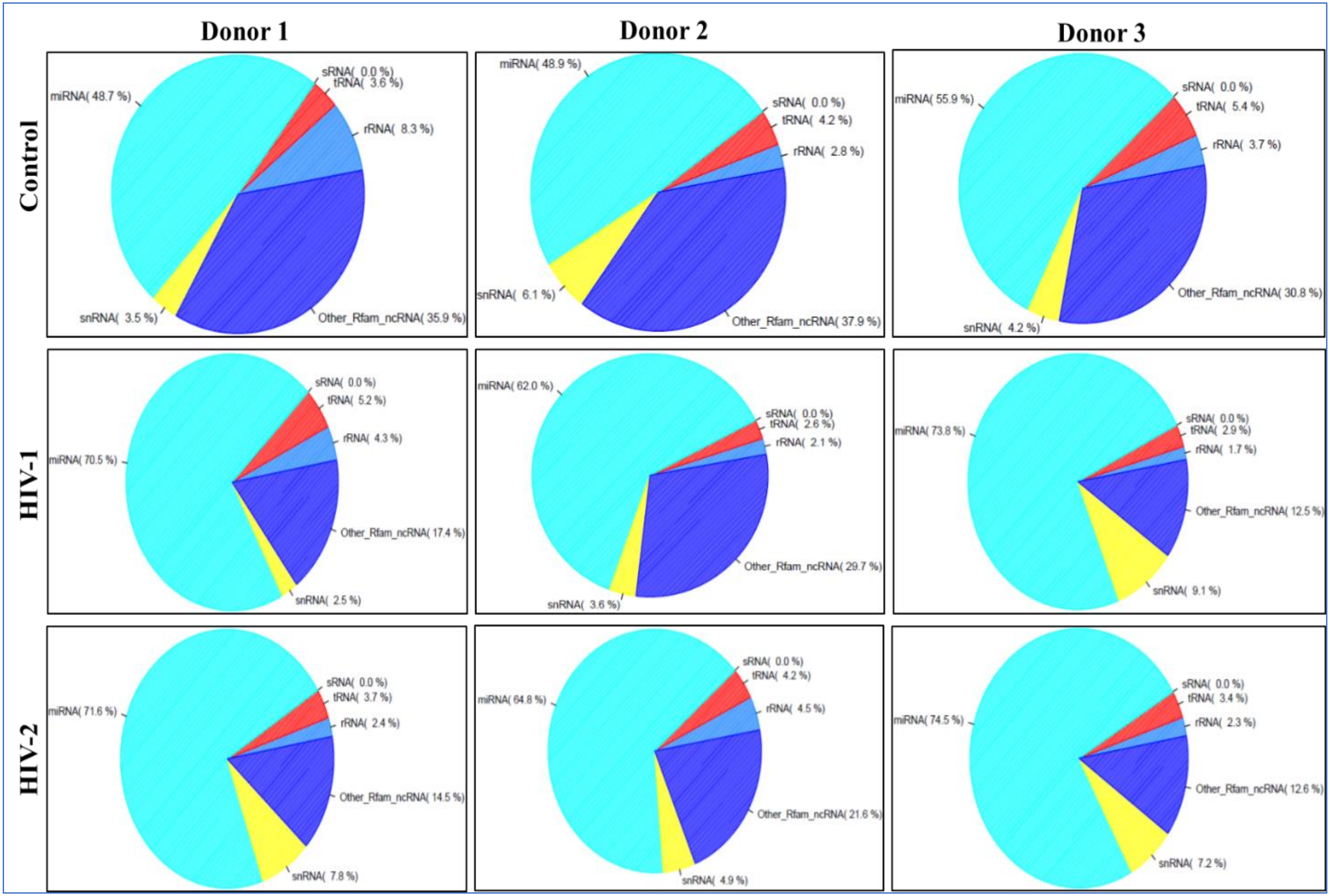
Donor 1
Donor 2
Donor 3
Control
HIV-1
HIV-2
miRNA(48.7 %)
sRNA(0.0 %)
tRNA(3.6 %)
rRNA(8.3 %)
snRNA(3.5 %)
Other_Rfam_ncRNA(35.9 %)
miRNA(48.9 %)
sRNA(0.0 %)
tRNA(4.2 %)
rRNA(2.8 %)
snRNA(6.1 %)
Other_Rfam_ncRNA(37.9 %)
miRNA(55.9 %)
sRNA(0.0 %)
tRNA(5.4 %)
rRNA(3.7 %)
Other_Rfam_ncRNA(30.8 %)
snRNA(4.2 %)
sRNA(0.0 %)
tRNA(5.2 %)
rRNA(4.3 %)
miRNA(70.5 %)
Other_Rfam_ncRNA(17.4 %)
snRNA(2.5 %)
miRNA(62.0 %)
sRNA(0.0 %)
tRNA(2.6 %)
rRNA(2.1 %)
Other_Rfam_ncRNA(29.7 %)
snRNA(3.6 %)
sRNA(0.0 %)
tRNA(2.9 %)
miRNA(73.8 %)
rRNA(1.7 %)
Other_Rfam_ncRNA(12.5 %)
snRNA(9.1 %)
sRNA(0.0 %)
tRNA(3.7 %)
miRNA(71.6 %)
rRNA(2.4 %)
Other_Rfam_ncRNA(14.5 %)
snRNA(7.8 %)
sRNA(0.0 %)
tRNA(4.2 %)
miRNA(64.8 %)
rRNA(4.5 %)
Other_Rfam_ncRNA(21.6 %)
snRNA(4.9 %)
sRNA(0.0 %)
tRNA(3.4 %)
miRNA(74.5 %)
rRNA(2.3 %)
Other_Rfam_ncRNA(12.6 %)
snRNA(7.2 %)

(A) MDMs

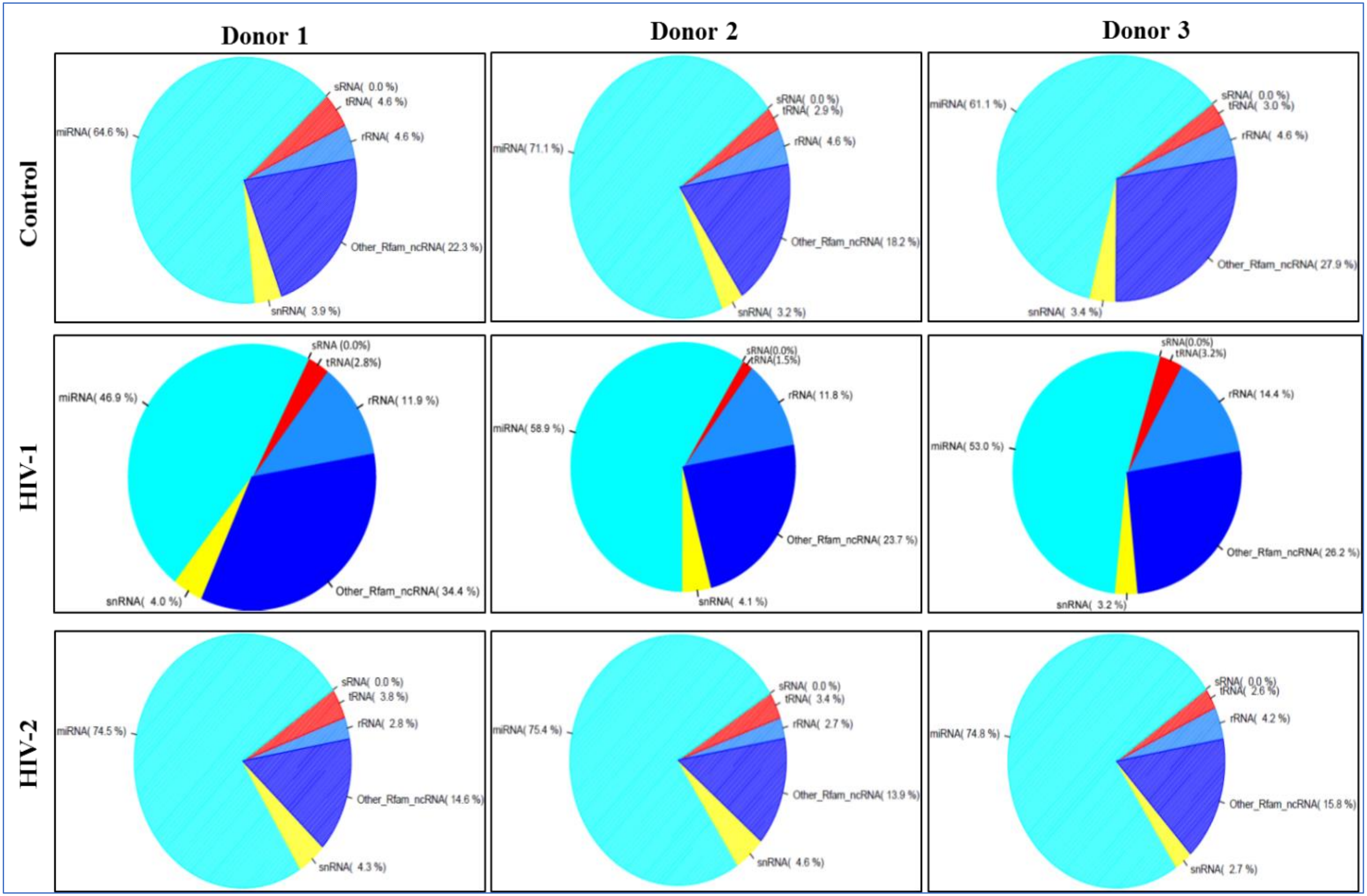

Donor 1
Donor 2
Donor 3
Control
HIV-1
HIV-2
sRNA(0.0 %)
tRNA(4.6 %)
rRNA(4.6 %)
miRNA(64.6 %)
Other_Rfam_ncRNA(22.3 %)
snRNA(3.9 %)
sRNA(0.0 %)
tRNA(2.9 %)
rRNA(4.6 %)
miRNA(71.1 %)
Other_Rfam_ncRNA(18.2 %)
snRNA(3.2 %)
sRNA(0.0 %)
tRNA(3.0 %)
rRNA(4.6 %)
miRNA(61.1 %)
Other_Rfam_ncRNA(27.9 %)
snRNA(3.4 %)
sRNA (0.0%)
tRNA(2.8%)
rRNA(11.9 %)
miRNA(46.9 %)
Other_Rfam_ncRNA(34.4 %)
snRNA(4.0 %)
sRNA(0.0%)
tRNA(1.5%)
rRNA(11.8 %)
miRNA(58.9 %)
Other_Rfam_ncRNA(23.7 %)
snRNA(4.1 %)
sRNA(0.0%)
tRNA(3.2%)
rRNA(14.4 %)
miRNA(53.0 %)
Other_Rfam_ncRNA(26.2 %)
snRNA(3.2 %)
sRNA(0.0 %)
tRNA(3.8 %)
rRNA(2.8 %)
miRNA(74.5 %)
Other_Rfam_ncRNA(14.6 %)
snRNA(4.3 %)
sRNA(0.0 %)
tRNA(3.4 %)
rRNA(2.7 %)
miRNA(75.4 %)
Other_Rfam_ncRNA(13.9 %)
snRNA(4.6 %)
sRNA(0.0 %)
tRNA(2.6 %)
rRNA(4.2 %)
miRNA(74.8 %)
Other_Rfam_ncRNA(15.8 %)
snRNA(2.7 %)

(B) PBMCs

Figure S2: **Frequency of different classes of RNA in small RNA libraries**.

The pie-charts represent an overview of small RNA expression in (A) uninfected control MDMs and HIV-1/HIV-2 infected MDMs; (B) uninfected control PBMCs and HIV-1/HIV-2 infected PBMCs.
