# Supplementary material for: Comparison of miRNA Expression Profiles between HIV-1 and HIV-2 Infected Monocyte-Derived Macrophages (MDMs) and Peripheral Blood Mononuclear Cells (PBMCs)
Source: Int J Mol Sci. 2020 Sep 22;21(18):6970. doi: 10.3390/ijms21186970 (PMC7556008; doi:10.3390/ijms21186970)
Supplement: Supplementary file 1 [file ijms-21-06970-s001.zip › Figure S3.pdf]

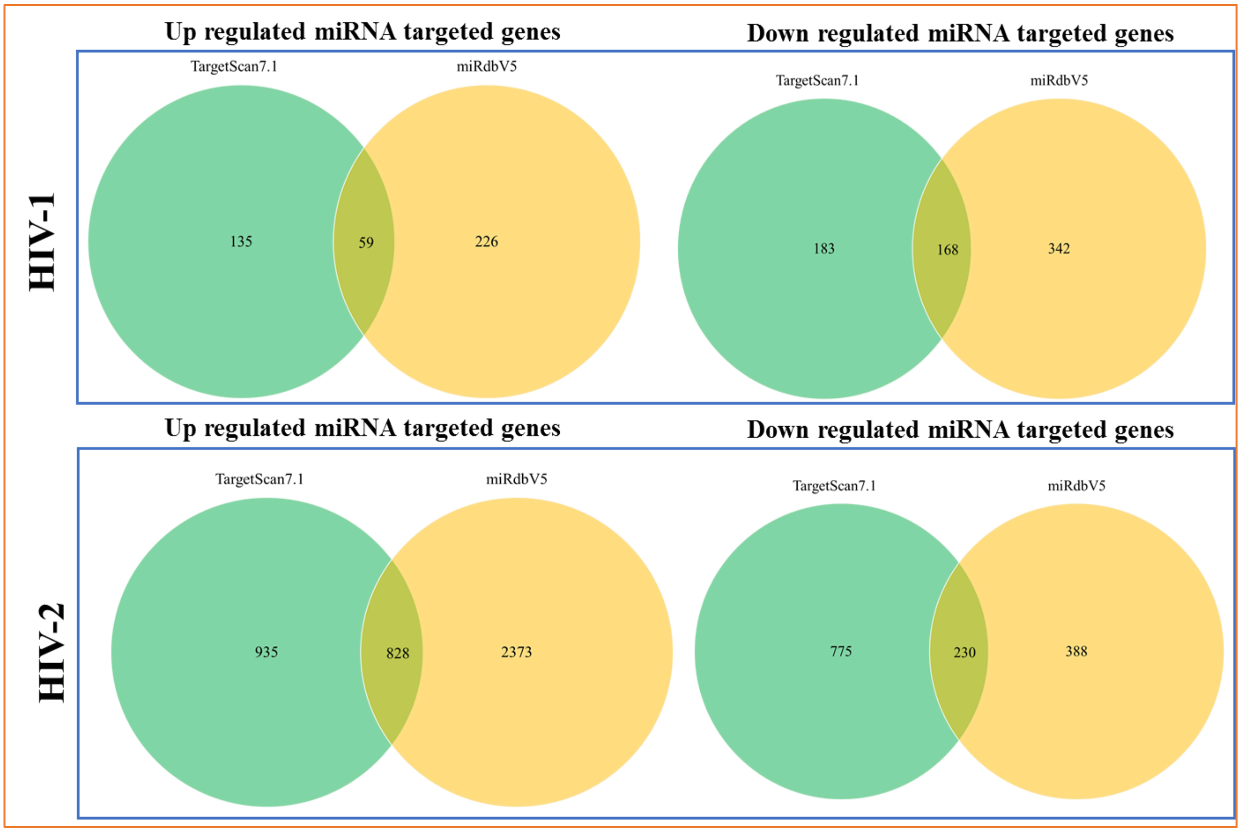

(A) MDMs

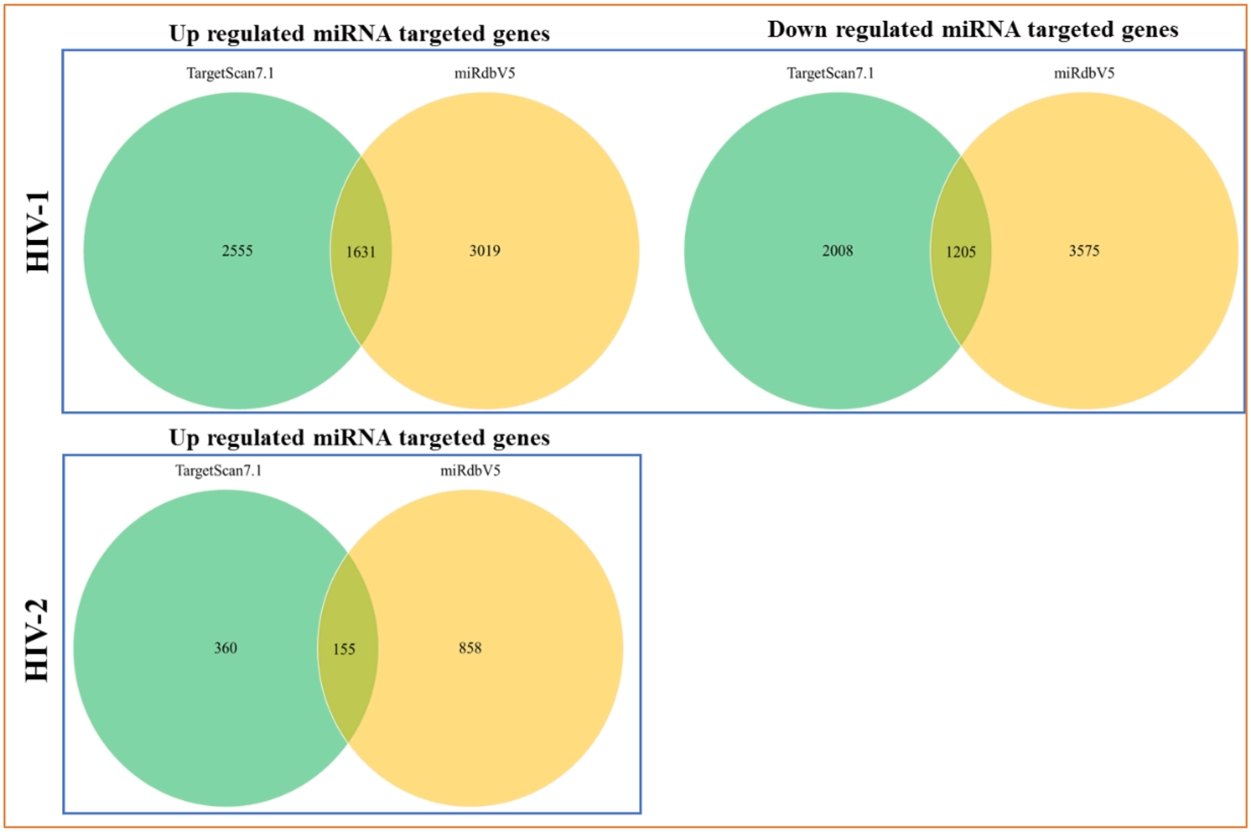

(B) PBMCs

Figure S3: Venn diagram representation of the number of differentially expressed miRNA targeted genes identified by TagetScan 7.1 and miRdbV5 database that are overlapping among HIV-1/ HIV-2 infected **MDMs (A) and PBMCs (B)** compared with uninfected control MDMs and PBMCs
