# Supplementary material for: Comparison of miRNA Expression Profiles between HIV-1 and HIV-2 Infected Monocyte-Derived Macrophages (MDMs) and Peripheral Blood Mononuclear Cells (PBMCs)
Source: Int J Mol Sci. 2020 Sep 22;21(18):6970. doi: 10.3390/ijms21186970 (PMC7556008; doi:10.3390/ijms21186970)
Supplement: Supplementary file 1 [file ijms-21-06970-s001.zip › Figure S4.pdf]

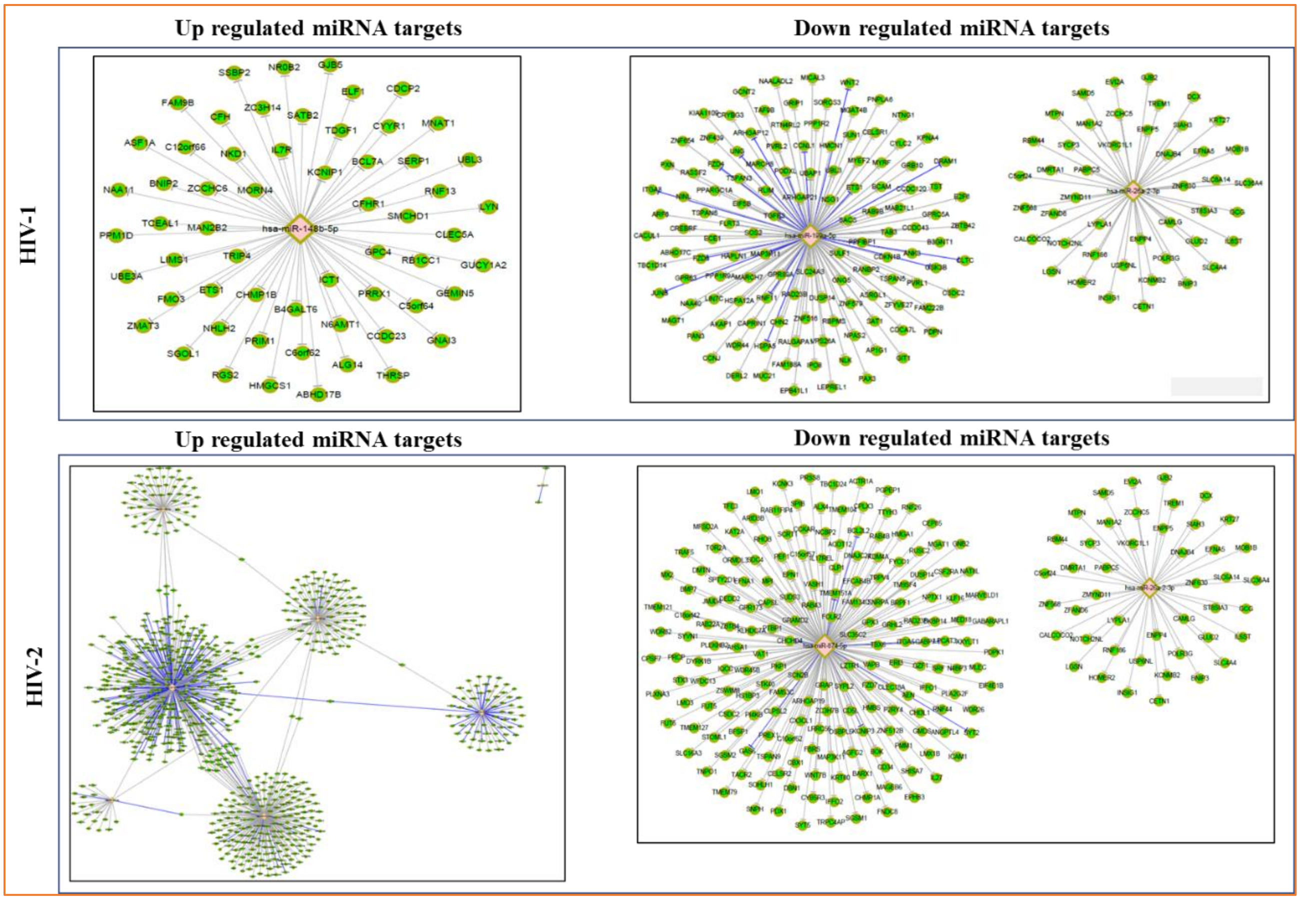

(A) MDMs

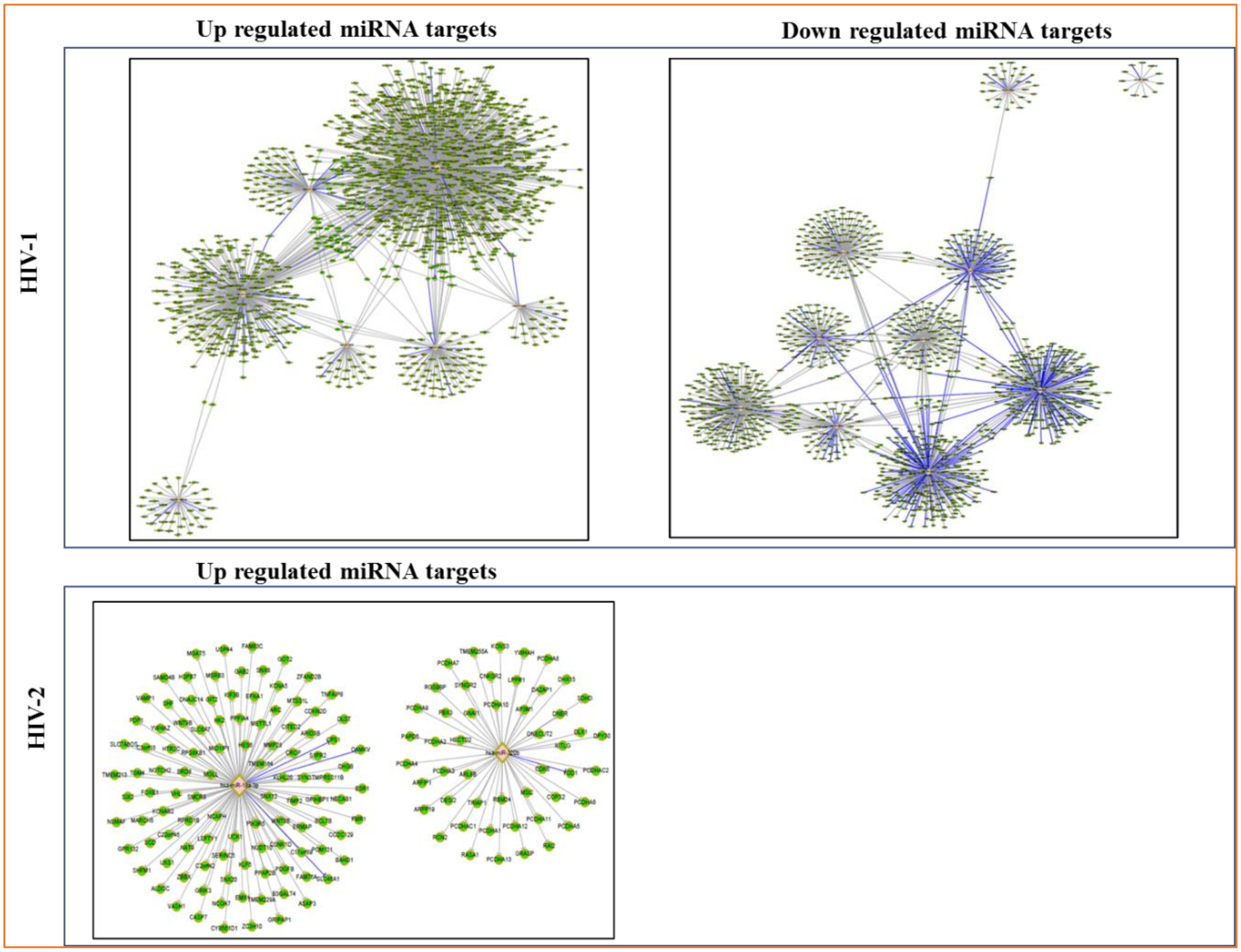

(B) PBMCs

Figure S4: **Network of putative interactions between miRNAs and their target genes related to HIV-1/HIV-2 infection in MDMs (A) and PBMCs (B).** Visualization of miRNAs and their associated target genes network with Cytoscape. The interaction network shows nodes and connections between miRNAs and the target genes. The Pink diamond nodes represent the miRNA and green circle nodes represent its targeted gene. The gray lines represent targets not experimentally validated, blue lines represent targets that are experimentally validated.
