# Supplementary material for: Comparison of miRNA Expression Profiles between HIV-1 and HIV-2 Infected Monocyte-Derived Macrophages (MDMs) and Peripheral Blood Mononuclear Cells (PBMCs)
Source: Int J Mol Sci. 2020 Sep 22;21(18):6970. doi: 10.3390/ijms21186970 (PMC7556008; doi:10.3390/ijms21186970)
Supplement: Supplementary file 1 [file ijms-21-06970-s001.zip › Figure S5.pdf]

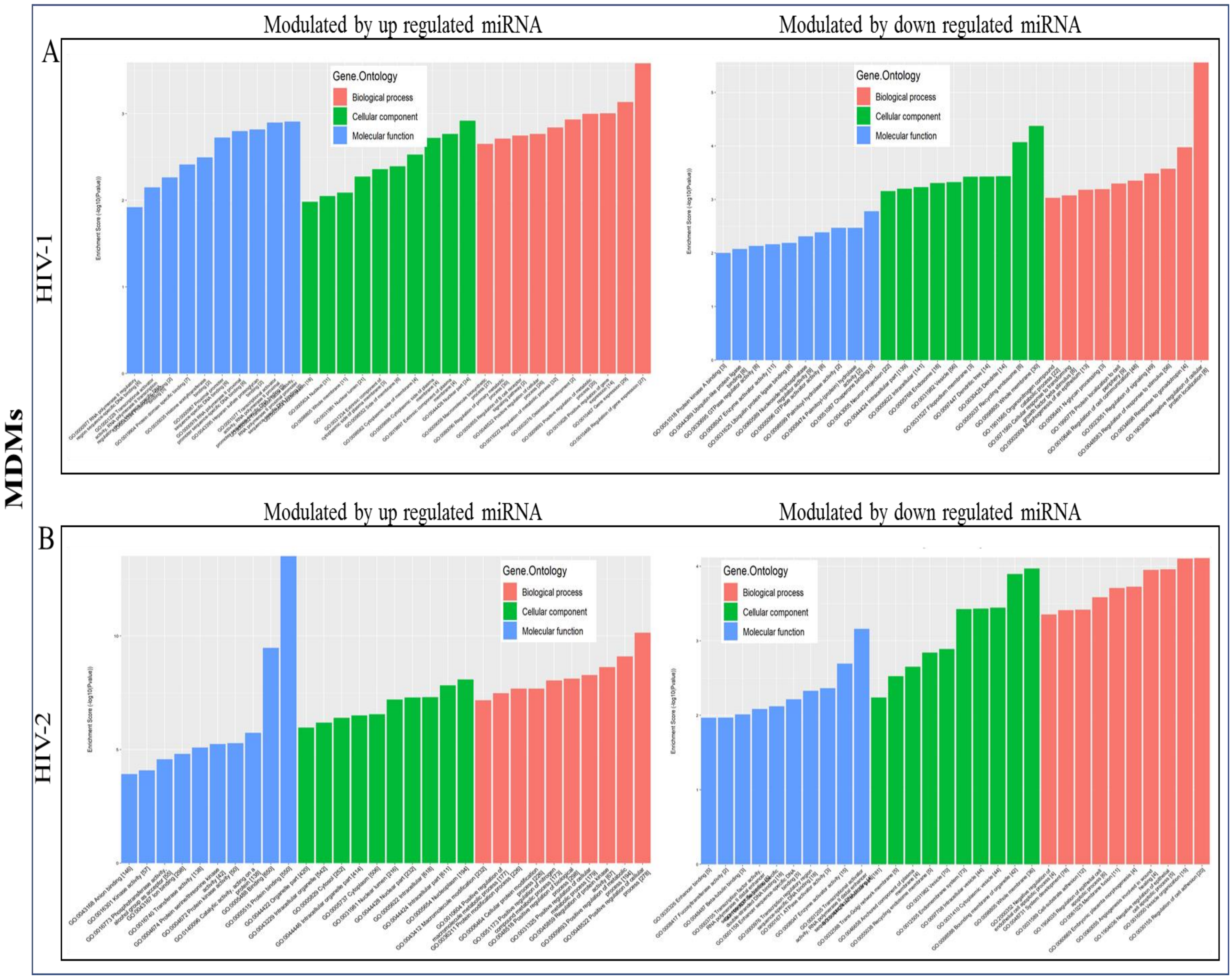
MDMs
A
HIV-1
Modulated by up regulated miRNA
Modulated by down regulated miRNA
B
HIV-2
Modulated by up regulated miRNA
Modulated by down regulated miRNA
Gene.Ontology
Biological process
Cellular component
Molecular function
Enrichment Score (-log10(Pvalue))

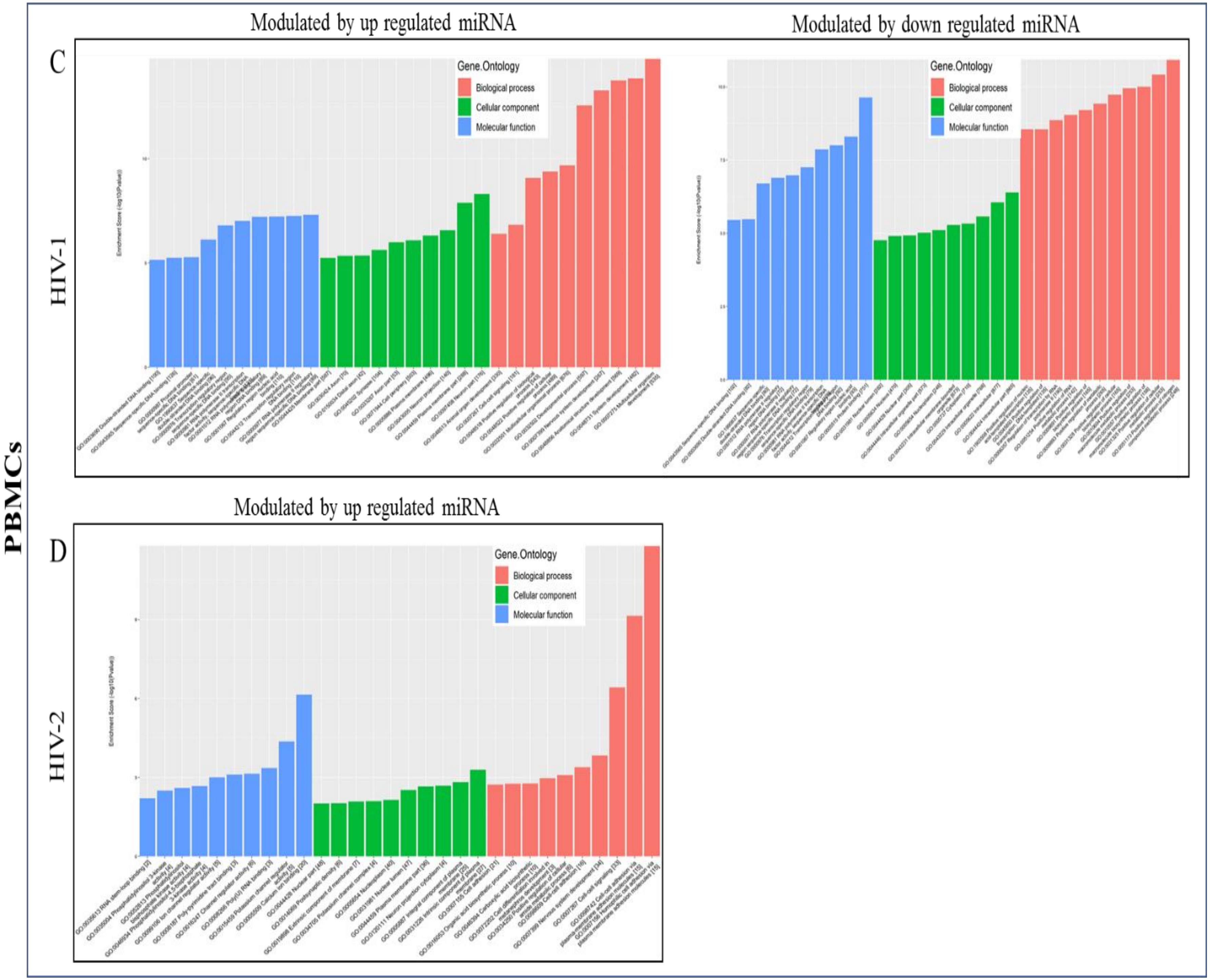
PBMCs
Modulated by up regulated miRNA
Modulated by down regulated miRNA
C
HIV-1
Gene.Ontology
Biological process
Cellular component
Molecular function
Gene.Ontology
Biological process
Cellular component
Molecular function
Modulated by up regulated miRNA
D
HIV-2
Gene.Ontology
Biological process
Cellular component
Molecular function

Figure S5: **GO analysis of differentially expressed miRNAs targets in MDMs and PBMCs.**

(A) GO functional annotation of differentially expressed miRNAs predicted target genes, including biological process, cellular component and molecular function in HIV-1 infected MDMs.

(B) GO functional annotation of differentially expressed miRNAs predicted target genes, including biological process, cellular component and molecular function in HIV-2 infected MDMs.

(C) GO functional annotation of differentially expressed miRNAs predicted target genes, including biological process, cellular component and molecular function in HIV-1 infected PBMCs.

(D) GO functional annotation of differentially expressed miRNAs predicted target genes, including biological process, cellular component and molecular function in HIV-2 infected PBMCs.
